# Smartphone‐Guided Visible Light Modulation of RSNOs for Antimicrobial Activity via Controlled Nitric Oxide Release

**DOI:** 10.1002/advs.77846

**Published:** 2026-09-27

**Authors:** Alexander Bruckmann, Adam Brooks Goodman, Myddelton C. Parker, Mark Garren, Ji Eun Park, Chad Schmiedt, Hitesh Handa, Elizabeth J. Brisbois

**Affiliations:** ^1^ School of Chemical Materials and Biomedical Engineering College of Engineering University of Georgia Athens Georgia USA; ^2^ Department of Small Animal Medicine and Surgery College of Veterinary Medicine University of Georgia Athens Georgia USA; ^3^ Department of Pharmaceutical and Biomedical Sciences College of Pharmacy University of Georgia Athens Georgia USA

**Keywords:** antimicrobial, antimicrobial blue light, controlled release, nitric oxide

## Abstract

Methicillin‐resistant *Staphylococcus aureus* (MRSA) remains a major contributor to antimicrobial‐resistant infections worldwide. Antimicrobial blue light (aBL, ∼405‐450 nm) generates intracellular reactive oxygen species through excitation of endogenous chromophores, while nitric oxide (NO) induces complementary nitrosative and oxidative damage. *S*‐nitrosothiols (RSNOs) provide a platform for visible‐light‐triggered NO release. Here, a synergistic antimicrobial strategy combining aBL with RSNO coatings was developed to simultaneously generate reactive oxygen and nitrogen species and evaluated against bacterial and fungal pathogens. Four RSNO donors (*S*‐nitroso‐*N*‐acetylpenicillamine (SNAP), *S*‐nitrosoglutathione (GSNO), *S*‐nitroso‐1‐adamantanethiol (SNAT), and *S*‐nitrosotriphenylmethanethiol (Ph_3_‐CSNO)) were incorporated into catheter insert coatings and exhibited distinct, wavelength‐dependent NO‐release profiles, with aBL enhancing NO release for all formulations. SNAT showed the greatest photochemical response, with a 235% increase in steady‐state NO flux under irradiation. The combined generation of reactive species produced potent broad‐spectrum antimicrobial activity against *Staphylococcus aureus*, MRSA, *Escherichia coli*, and *Candida albicans*, achieving >4‐log reductions in microbial viability with all RSNO‐coated inserts under aBL after 24 h. In vivo, SNAT‐coated inserts significantly reduced *S. aureus* colonization in a rabbit infection model, achieving up to a 2.35‐log reduction in bacterial burden, demonstrating that light‐triggered NO delivery combined with aBL provides an antibiotic‐independent strategy for preventing device‐associated infections.

## Graphical abstract

A light‐activated catheter insert combines antimicrobial blue light with nitric oxide release to generate complementary oxidative and nitrosative stress, producing potent broad‐spectrum antimicrobial activity. The approach achieves greater than 4‐log reductions in bacterial and fungal viability and significantly reduces *Staphylococcus aureus* colonization in vivo, providing a promising antibiotic‐independent strategy for preventing catheter‐associated infections.

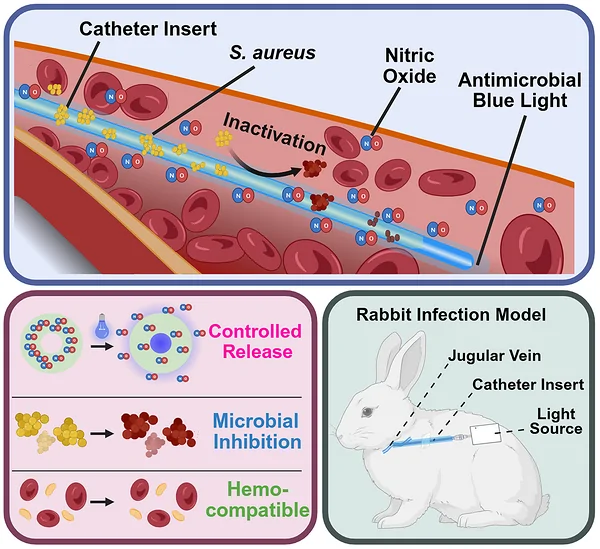

## 1 Introduction

Worldwide, approximately 138 million hospitalized patients acquire infections each year caused by antimicrobial‐resistant (AMR) pathogens, including *Escherichia coli* (*E. coli*) and *Staphylococcus aureus* (*S. aureus*) [1]. These infections were associated with an estimated 4.71 million deaths globally in 2021, and AMR‐related mortality is projected to nearly double by 2050 [2, 3]. Consequently, there is an urgent need to develop alternative antimicrobial agents, biomaterials, and therapeutic strategies that prevent and treat infections without accelerating the emergence of resistance. In particular, methicillin‐resistant *S. aureus* (MRSA) remains a major clinical concern, as MRSA infections are associated with a substantially greater risk of mortality than infections caused by methicillin‐sensitive strains [4].

A key virulence factor produced by *S. aureus* is the carotenoid pigment staphyloxanthin (STX), which functions as a membrane‐bound antioxidant, protecting bacterial cells from reactive oxygen species (ROS) and immune‐mediated killing [5]. As a result, strategies that increase oxidative stress or disrupt STX‐mediated protection have emerged as promising approaches for combating MRSA infections. One such strategy is antimicrobial blue light (aBL, λ = 400–470 nm), which generates ROS through photoexcitation of endogenous microbial chromophores [6]. Among visible wavelengths, aBL exhibits particularly strong antimicrobial activity and has demonstrated efficacy against bacterial and fungal pathogens, including *E. coli*, *S. aureus*, and *Candida albicans* (*C. albicans*) [7, 8, 9]. Importantly, aBL does not appear to promote antimicrobial resistance, making it a suitable alternative for infection prevention and control [8, 10].

Nitric oxide (NO) is another promising non‐antibiotic antimicrobial agent that exerts broad‐spectrum activity through the formation of reactive nitrogen species (RNS) and ROS capable of damaging microbial membranes, proteins, and nucleic acids [11]. Due to its short half‐life, various NO donor molecules have been developed to enable controlled NO delivery [12]. Among these, *S*‐nitrosothiols (RSNOs) are particularly attractive because they undergo photolytic cleavage under visible light, enabling externally triggered and tunable NO release [13]. Different RSNOs, including *S*‐nitroso‐*N*‐acetylpenicillamine (SNAP), *S*‐nitrosoglutathione (GSNO), *S*‐nitroso‐1‐adamantanethiol (SNAT), and *S*‐nitrosotriphenylmethanethiol (Ph_3_‐CSNO), exhibit distinct molecular structures that can have a direct impact on their stability and release characteristics, which may influence their antimicrobial performance. The ability to combine aBL with light‐triggered NO release offers a multifunctional antimicrobial approach in which ROS, RNS, and NO act in combination to enhance microbial killing. While previous studies have reported enhanced antimicrobial activity through the combination of light irradiation and NO release, systematic evaluation of wavelength‐dependent RSNO activation, donor‐dependent release behavior, the resulting antimicrobial efficacy across diverse microbial species, and translation of these strategies to in vivo infection models remains limited [14, 15].

**SCHEME 1:**
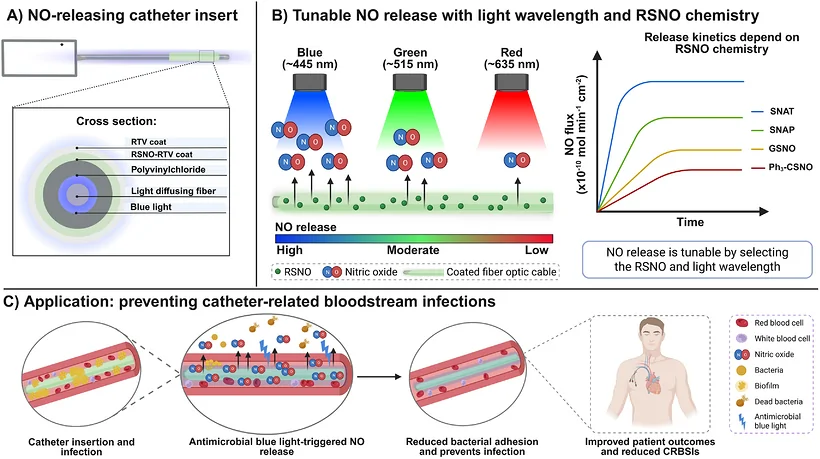
Overview of the light‐responsive nitric oxide (NO)‐releasing catheter insert and its antimicrobial mechanism. (A) Schematic of the NO‐releasing catheter insert consisting of a blue light‐diffusing optical fiber surrounded by a poly(vinyl chloride) (PVC) catheter and an *S*‐nitrosothiol (RSNO)‐functionalized silicone (RTV) coating. (B) Blue, green, and red light irradiation activate photolytic NO release from different RSNO chemistries, enabling tunable NO release kinetics and total NO payload through selection of both the light wavelength and RSNO donor. (C) Proposed application for preventing catheter‐related bloodstream infections (CRBSIs), where blue light‐triggered NO release inhibits bacterial adhesion and biofilm formation on the catheter surface, reducing bacterial colonization and subsequent infection.

To investigate whether combining NO release with aBL irradiation can enhance antimicrobial activity to prevent catheter‐associated infections, NO‐releasing catheter inserts (CIs) integrated with fiber‐optic light delivery were developed (Scheme 1). This design provides a clinically relevant strategy for delivering light to catheters, enabling repeated or continuous activation of NO release during device use without interrupting therapy or requiring catheter exchange. Primarily, the CIs were developed as an antibiotic‐free strategy for preventing and reducing infection associated with indwelling catheters, including peripheral intravenous catheters, while providing a potentially noninvasive approach for tunneled, blood‐contacting catheters such as central venous catheters. Upon aBL irradiation, photolysis of incorporated RSNO donors triggers NO release and promotes photochemical processes that contribute to ROS generation, thereby providing a multimodal antimicrobial mechanism. Four RSNO donors, SNAP, GSNO, SNAT, and Ph_3_‐CSNO, were synthesized and incorporated (20 wt%) into Room Temperature Vulcanizing (RTV)‐based silicone coatings on side‐glowing fiber optic CIs using a dip‐coating approach. The resulting materials were characterized for wavelength‐dependent NO release, donor leaching, and the production of reactive nitrogen and oxygen species, including nitrite and hydrogen peroxide equivalents, under physiological conditions. Antimicrobial efficacy was evaluated against *S. aureus*, MRSA, *E. coli*, and *C. albicans*. In addition, cytocompatibility was assessed using L‐929 mouse fibroblasts, while hemocompatibility was evaluated through hemolysis and platelet adhesion studies. Finally, the lead CI formulation was assessed in a 7‐h rabbit catheter infection model challenged with *S. aureus* to evaluate its ability to reduce bacterial colonization under physiologically relevant conditions. This in vivo evaluation further distinguishes the present platform from prior studies focused primarily on in vitro characterization of light‐activated NO‐releasing catheter materials.

## 2 Results

### 2.1 Fabrication of RSNO‐Coated CIs

**FIGURE 1:**
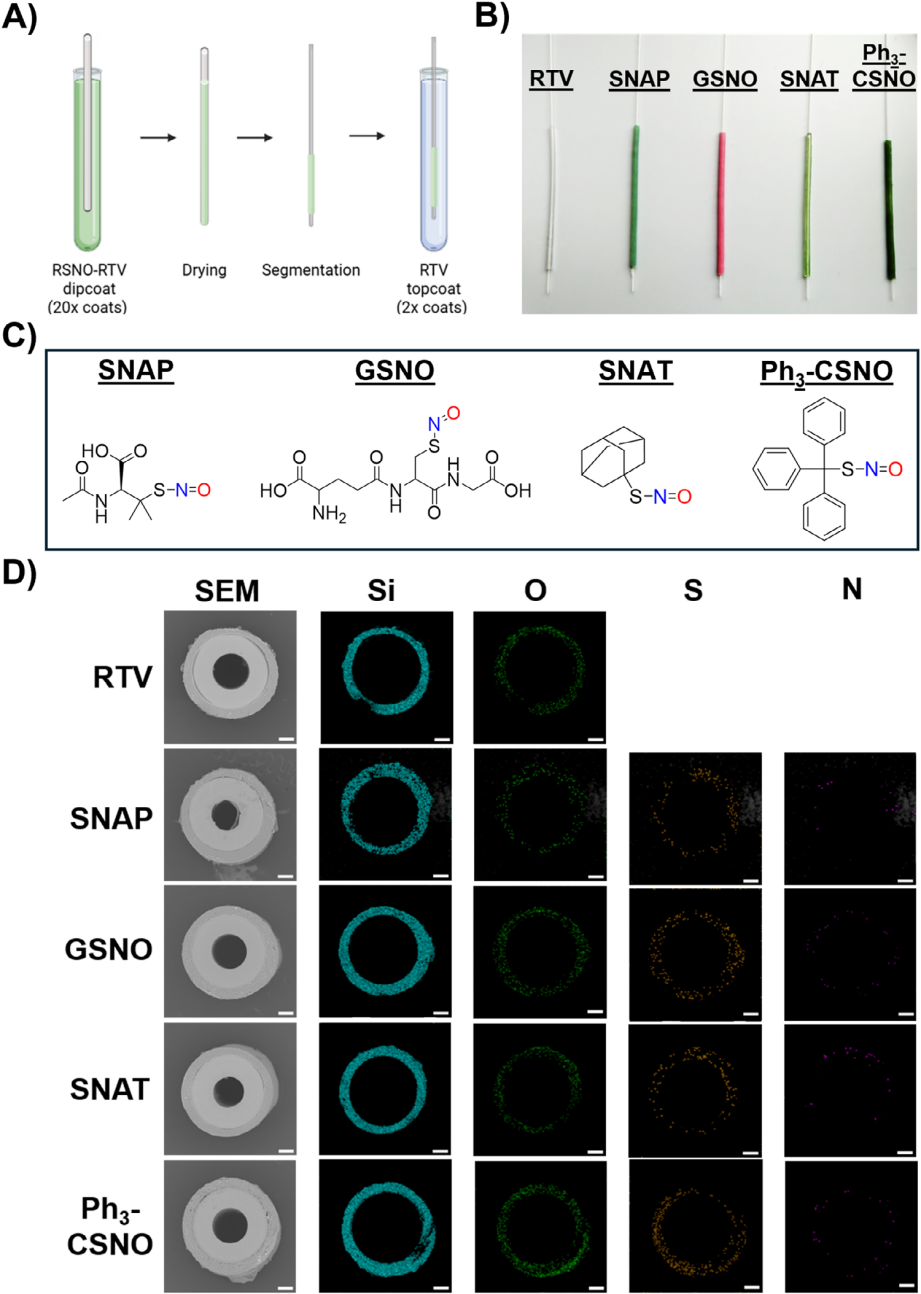
(A) Fabrication schematic of RSNO‐coated CIs via a dip‐coating method using RTV as the base polymer. The RSNO‐RTV solution was coated onto PVC jackets (20x) with 1 min drying in between, allowed to dry overnight, and cut to 3 cm before sealing with 2 topcoats of RTV. (B) Representative images of RSNO‐coated CIs (3 cm in length) with 2 coats of RTV. (C) Chemical structures of the RSNOs. (D) SEM cross‐sectional images and EDS elemental mapping of RTV and RSNO‐coated CIs. RSNO‐coated samples exhibited uniform coatings (∼150 µm thick) with sulfur and nitrogen signals indicative of RSNO incorporation, whereas no sulfur or nitrogen was detected in RTV controls. Scale bars correspond to 200 µm.

To investigate how the molecular structure of different RSNOs influences visible light‐triggered NO release and the resulting antimicrobial activity, catheter inserts (CIs) incorporating four chemically distinct RSNOs were fabricated. To generate the NO‐releasing CIs, poly(vinyl chloride) (PVC) jacket segments were dip‐coated in an RSNO‐RTV‐THF solution (100 mg mL^−1^ RTV containing 20 wt% RSNO), followed by application of an RTV topcoat (Figure 1A). Dip coating was selected as it enables reproducible deposition of polymer coatings with relatively uniform thickness on cylindrical medical‐device substrates and has been widely employed for the fabrication of NO‐releasing catheters and extracorporeal circuit components [16, 17, 18].

Uniform incorporation of the NO donor throughout the polymer matrix contributes to consistent coating morphology and more predictable NO‐release behavior [19]. In addition, the RTV topcoat acts as a protective diffusion barrier, creating a smoother surface, and has been shown to prolong NO release by reducing donor leaching and limiting environmental exposure of the NO donor reservoir [20]. The topcoat also facilitated secure attachment of the catheter insert to the fiber optic, improving fabrication consistency and reproducibility between samples (Figure 1B). A donor loading of 20 wt% was selected as it produced uniform coatings across all four RSNO formulations while maintaining solution processability during dip coating.

Higher donor loadings resulted in poor coating quality for GSNO due to its limited solubility in THF. Furthermore, preliminary optimization studies indicated that lower donor loadings were unable to consistently achieve physiologically relevant endothelial NO fluxes (0.5–4.0 × 10^−10^ mol min^−1^ cm^−2^) for multiple days [21]. Microscopy analysis via SEM and EDS confirmed successful coating deposition, with all RSNO‐coated CIs exhibiting uniform coatings approximately 150 µm thick and detectable sulfur and nitrogen signals indicative of RSNO incorporation. In contrast, sulfur and nitrogen were absent from the RTV‐coated control inserts (Figure 1C).

### 2.2 Photo‐Triggered No Release

**FIGURE 2:**
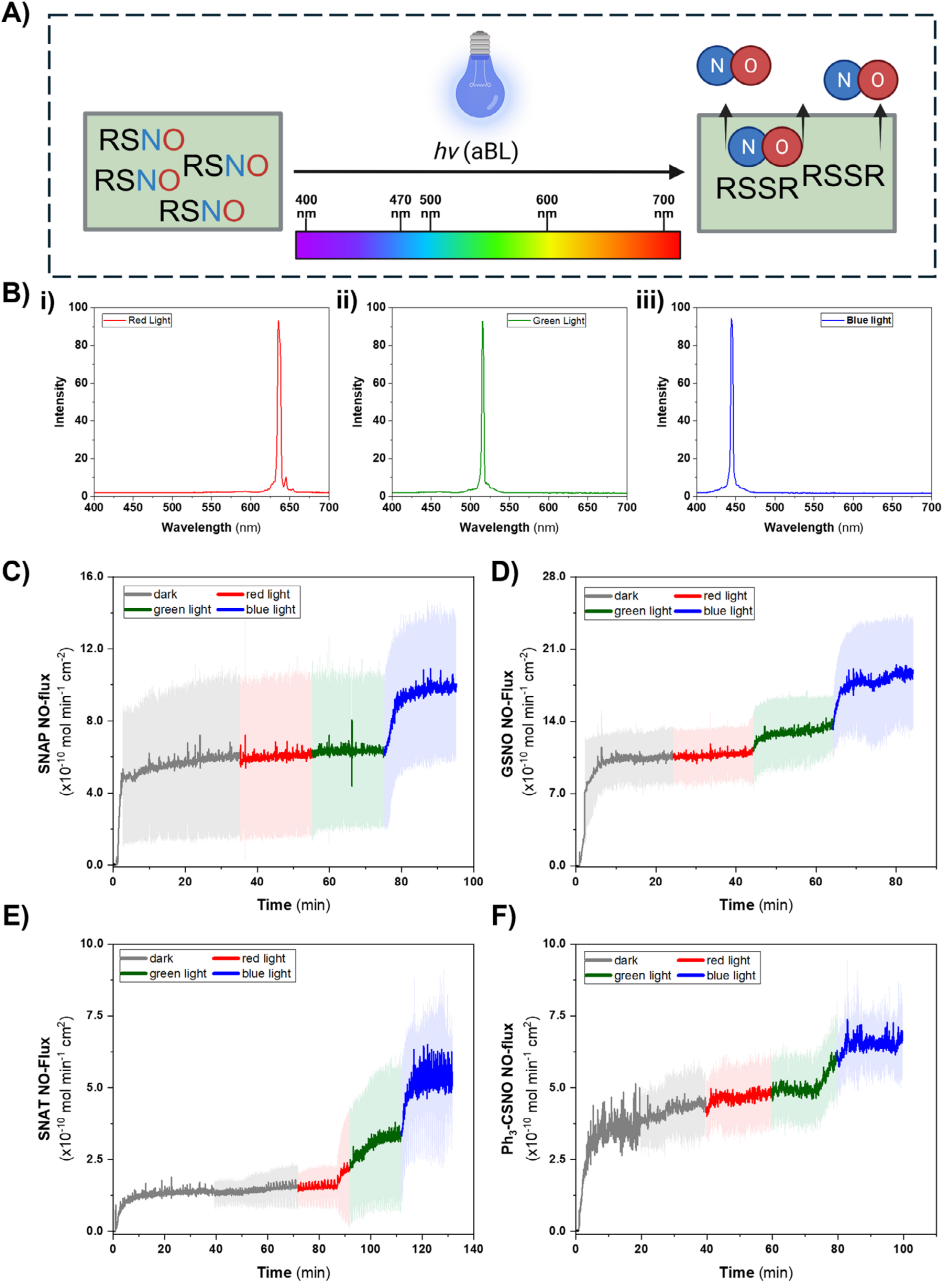
Wavelength‐dependent NO release. (A) Release of NO from RSNOs via visible light irradiation, including aBL. (B) Emission spectra of the i) red, ii) green, and iii) blue LEDs used, exhibiting peak wavelengths of 635, 515, and 445 nm, respectively. Real‐time measurements of steady‐state NO release from (C) SNAP‐, (D) GSNO‐, (E) SNAT‐, and (F) Ph_3_‐CSNO‐coated CIs under physiological conditions. Exposure to red, green, and blue light occurred after a steady‐state plateau was achieved under the preceding condition, beginning with no light exposure. Solid lines represent the mean NO release profile, and the shaded regions indicate the SD (*n* ≥ 5).

Visible light induces photolytic decomposition of RSNOs, resulting in the release of NO and formation of the corresponding disulfide (RSSR) (Figure 2A) [22]. To determine the effect of irradiation wavelength on NO release, coated CIs were evaluated under physiological conditions. Sequential irradiation was performed using red, green, and blue light at maximum intensity, with emission spectra centered at 635 nm, 515 nm, and 445 nm, respectively, as measured using a wireless spectrophotometer (Figure 2Bi‐iii). Each wavelength was applied sequentially for 20 min via smartphone app, and NO release was monitored until a new steady‐state flux was achieved before exposure to the next wavelength. Across all RSNOs, red light produced no change in NO release (Figure 2C–F).

Green light led to a modest increase in NO flux for GSNO and SNAT, whereas aBL induced a pronounced increase in NO release for all donors. Under dark conditions, baseline NO flux varied among the RSNOs, increasing in the order SNAT < Ph_3_‐CSNO < SNAP < GSNO. These differences are consistent with the predicted donor hydrophilicity and lipophilicity (Table 1), as more water‐soluble RSNOs (e.g., GSNO and SNAP) interact more strongly with the aqueous environment, thereby facilitating donor accessibility and NO release [23]. Upon aBL irradiation, NO flux increased for each RSNO; however, the magnitude of the enhancement was donor‐dependent, with increases of approximately 54.02%, 75.39%, 235.45%, and 63.22% observed for SNAP, GSNO, SNAT, and Ph_3_‐CSNO, respectively, compared to dark conditions.

**TABLE 1:** Predicted hydrophilicity/lipophilicity and theoretical NO loading of the RSNOs.

RSNO	log*P*	Hydrophilicity and lipophilicity	Total theoretical moles of NO (mol/cm^2^)
GSNO	−1.72	Hydrophilic	8.74 × 10^−6^
SNAP	0.77	Moderate lipophilic	1.19 × 10^−5^
SNAT	3.37	Lipophilic	1.15 × 10^−5^
Ph_3_‐CSNO	5.39	Lipophilic	5.65 × 10^−5^

While all samples were fabricated using a consistent RSNO loading (20 wt% relative to the RTV polymer, w/w), the intrinsic molecular weight and structure of each RSNO result in differing theoretical molar NO loadings. Lower molecular weight RSNOs such as SNAP (220.24 g mol^−1^) and SNAT (197.30 g mol^−1^) therefore provide a higher theoretical NO donor content per unit mass than higher molecular weight RSNOs such as GSNO (336.32 g mol^−1^) and Ph_3_‐CSNO (305.40 g mol^−1^), which may contribute to differences in flux magnitude and release kinetics. These values were calculated based on the RSNO mass fraction and molecular weight with corresponding theoretical NO loadings normalized to coating surface area (Table 1). Importantly, these effects occur alongside differences in RSNO hydrophilicity and light sensitivity, which together govern the overall release behavior.

To further distinguish wavelength‐dependent photochemical efficiency from differences in NO flux, the apparent quantum yield of NO release was calculated for each RSNO under red, green, and blue light (Table S1). Red light produced the lowest apparent quantum yields across all RSNOs, whereas blue light generally produced the highest values. GSNO exhibited the greatest apparent quantum yield under blue light (6.27 ± 2.77%), while SNAP, SNAT, and Ph_3_‐CSNO exhibited apparent quantum yields of 3.21 ± 1.76%, 3.10 ± 1.61%, and 1.65 ± 0.61%, respectively. These findings indicate that the enhanced NO release observed under blue light reflects not only differences in incident photon energy but also wavelength‐ and donor‐dependent photochemical efficiency. However, because the calculation was based on incident optical power rather than the fraction of photons absorbed by each RSNO‐coated CI, these values represent apparent rather than absolute quantum yields.

To assess whether donor diffusion contributed to light‐induced NO release, RSNO leaching into PBS was quantified over time using UV–vis spectroscopy at 340 nm. Both SNAP and GSNO exhibited measurable diffusion from the CIs, with approximately 0.23 ± 0.01 and 0.11 ± 0.01 µg RSNO cm^−2^ of polymer released after 24 h, respectively (Figure S1). Under aBL irradiation, SNAP diffusion significantly decreased to 0.13 ± 0.03 µg cm^−2^, whereas GSNO diffusion remained unchanged. This behavior may reflect reaction‐diffusion effects influenced by donor solubility and residence within the polymer matrix. Although GSNO is more water‐soluble, it likely remains within the coating's hydrated domains, where aBL promotes in situ NO release rather than increasing donor leaching. In contrast, SNAP may localize near the polymer‐solution interface, where light‐induced decomposition occurs concurrently with diffusion, thereby reducing apparent leaching despite increased NO generation. Consistent with their lipophilic character, SNAT and Ph_3_‐CSNO exhibited little to no detectable diffusion into the surrounding buffer (Figure S2). The absence of detectable SNAT and Ph_3_‐CSNO diffusion also suggests limited exposure of the surrounding aqueous environment to the corresponding donor and potential photolysis products. Previous studies have reported that photolysis of SNAP and GSNO produces corresponding disulfides and radical intermediates, which may contribute to biological responses if released from the polymer matrix [24, 25]. Previous work with SNAT‐impregnated PVC similarly demonstrated sustained NO release from the polymer matrix over extended periods, while the authors identified SNAT leaching as an important consideration for future investigation [26]. However, the low diffusion observed for SNAT and Ph_3_‐CSNO suggests that these species and their decomposition products would be predominantly retained within the catheter insert. Accordingly, the substantial increase in NO flux observed for SNAT under aBL (235.45%) can be attributed exclusively to light‐triggered NO release from the immobilized donor rather than contributions from diffused RSNO. Differences in the photochemical liberation of NO among donors may also arise from variations in S‐N bond environment and molecular structure, potentially accounting for the divergent responses observed between SNAT and Ph_3_‐CSNO. Overall, aBL irradiation produced a significant, donor‐dependent enhancement in NO release, outperforming red and green light across all RSNOs tested.

To determine whether prolonged aBL irradiation contributed to RSNO decomposition through localized heating, the surface temperature of the RSNO‐coated CIs was monitored over 24 h using a thermal imaging camera (Seek Thermal). Minimal temperature changes were observed throughout the irradiation period, indicating that the enhanced NO release was primarily attributable to photolysis rather than temperature‐induced thermal decomposition (Figure S3).

**FIGURE 3:**
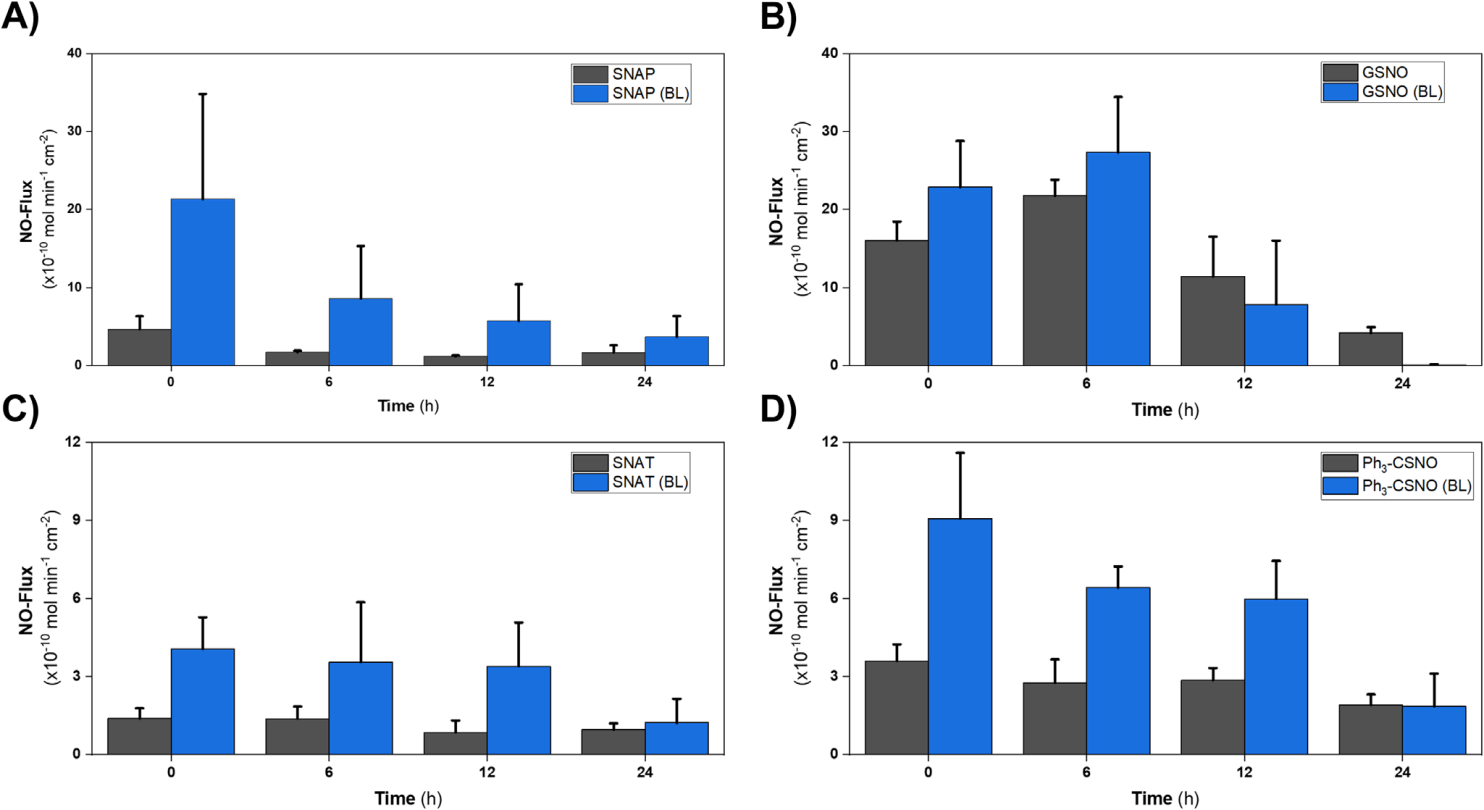
Average surface flux of NO released from SNAP‐ (A), GSNO‐ (B), SNAT‐ (C), and Ph_3_‐CSNO‐coated CIs (D) under physiological conditions over 24 h. Antimicrobial blue‐light (BL) samples were irradiated at each time point as well as irradiated continuously during incubation between measurements. Controls were tested and incubated in the absence of light. Data are shown as mean ± SD (*n ≥* 3).

Nitric oxide release from RSNO‐coated CIs was evaluated over a 24‐h period under physiological conditions to assess the effect of continuous blue light irradiation (Figure 3A–D). For each RSNO, aBL exposure resulted in elevated NO release at the initial (0 h) and 6 h time points relative to dark controls. By 24 h, NO flux from aBL‐treated samples was similar to controls for most RSNOs, whereas GSNO exhibited no detectable NO release, consistent with complete depletion of its available NO payload under sustained aBL irradiation. The SNAP and GSNO CIs displayed high initial NO release when irradiated with aBL, reaching 21.36 ± 13.44 × 10^−10 ^mol min^−1^ cm^−2^ and 22.87 ± 5.92 × 10^−10 ^mol min^−1^ cm^−2^, respectively, followed by a rapid decline, with SNAP stabilizing at lower flux levels by 6 h. In contrast, GSNO maintained comparatively higher NO release up to 6 h, likely reflecting its greater hydrophilicity (Table 1) and enhanced interaction with the aqueous environment. Both SNAT and Ph_3_‐CSNO exhibited lower initial flux values of 4.06 ± 1.22 × 10^−10 ^mol min^−1^ cm^−2^ and 9.08 ± 2.52 × 10^−10 ^mol min^−1^ cm^−2^, respectively; however, both formulations sustained NO release for up to 12 h before declining at 24 h, indicative of slower donor consumption. While differences in NO payload and donor structures likely contributed to these release profiles, all RSNO‐coated CIs demonstrated enhanced NO production under aBL irradiation over the 24 h evaluation period.

Additionally, NO release under blue‐, green‐, and red‐light irradiation was monitored for up to 7 days to evaluate long‐term release behavior and donor exhaustion (Figures S4–S7). Across all RSNO formulations, aBL consistently produced the greatest enhancement in NO release, whereas substantially lower responses were observed under green‐ and red‐light irradiation. The enhanced NO release observed under aBL irradiation is consistent with the photochemical behavior of RSNOs, which exhibit stronger absorption and more efficient S‐N bond cleavage at shorter visible wavelengths [27]. As a result, aBL provides higher‐energy photons capable of promoting RSNO photolysis more effectively than green or red light, leading to greater NO release. Previous studies investigating light‐triggered NO release from RSNO‐containing materials have similarly reported enhanced photolytic NO release under shorter‐wavelength visible light compared to longer wavelengths [15]. Therefore, aBL was selected for all subsequent experiments.

### 2.3 Production of Various RNS and ROS Species

Nitric oxide‐releasing compounds are known to participate in secondary redox reactions that can generate additional RNS and ROS, including nitrite and hydrogen peroxide, particularly under physiological conditions [28]. Light irradiation has also been shown to promote ROS formation through photoinduced redox processes, further contributing to oxidative and nitrosative stress [29]. These species play important roles in antimicrobial activity by contributing to oxidative damage and disruption of bacterial cellular function [14]. Accordingly, the production of nitrite and H_2_O_2_ equivalents from RSNO‐coated CIs was evaluated to better understand the broader reactive species profile associated with light‐triggered NO release.

**FIGURE 4:**
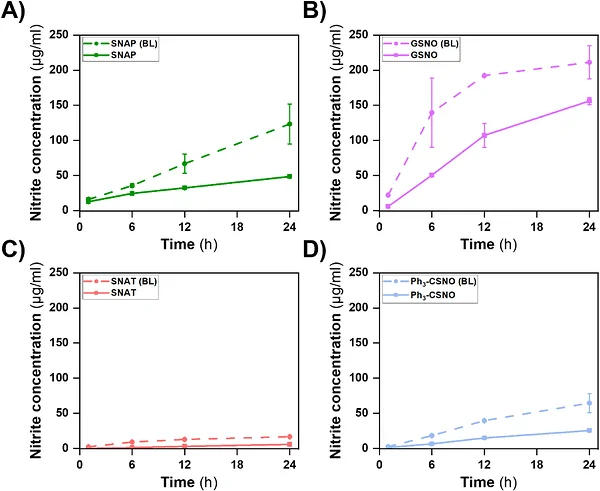
Average nitrite production from RSNO‐coated catheter inserts over 24 h. Increase in nitrite production with aBL irradiation from SNAP‐ (A), GSNO‐ (B), SNAT‐ (C), and Ph_3_‐CSNO‐coated CIs (D) under physiological conditions over 24 h. Antimicrobial blue light samples were irradiated throughout the study. Data are shown as mean ± SD (*n ≥* 3).

Nitrite generation from RSNO‐coated CIs was quantified using the Griess assay to assess downstream nitrogen oxide chemistry associated with NO release. Under dark conditions, all RSNOs exhibited time‐dependent nitrite formation, with donor‐dependent differences in magnitude. Upon aBL irradiation, nitrite production increased significantly for all RSNOs at each time point after 6 h when compared to respective controls (no light). At 24 h, SNAP exhibited an increase from approximately 48.57 ± 1.88 µg mL^−1^ under dark conditions to 123.43 ± 28.52 µg mL^−1^ with aBL (154.15% increase) (Figure 4A), while GSNO increased from 156.22 ± 5.04 µg mL^−1^ to 211.27 ± 23.48 µg mL^−1^ (35.23% increase) (Figure 4B). Similar to SNAP, a more profound increase was observed with Ph_3_‐CSNO, reaching ∼64.56 ± 13.53 µg mL^−1^ compared to 25.48 ± 0.38 µg mL^−1^ without aBL (153.38% increase) (Figure 4C). The largest relative enhancement among the donors was SNAT, increasing from 5.83 ± 0.25 µg mL^−1^ to 18.79 ± 0.56 µg mL^−1^, corresponding to an approximately 188.19% increase (Figure 4D). Across all conditions, nitrite production followed the trend SNAT < Ph_3_‐CSNO < SNAP < GSNO, consistent with the donor‐dependent NO release profiles observed previously.

**FIGURE 5:**
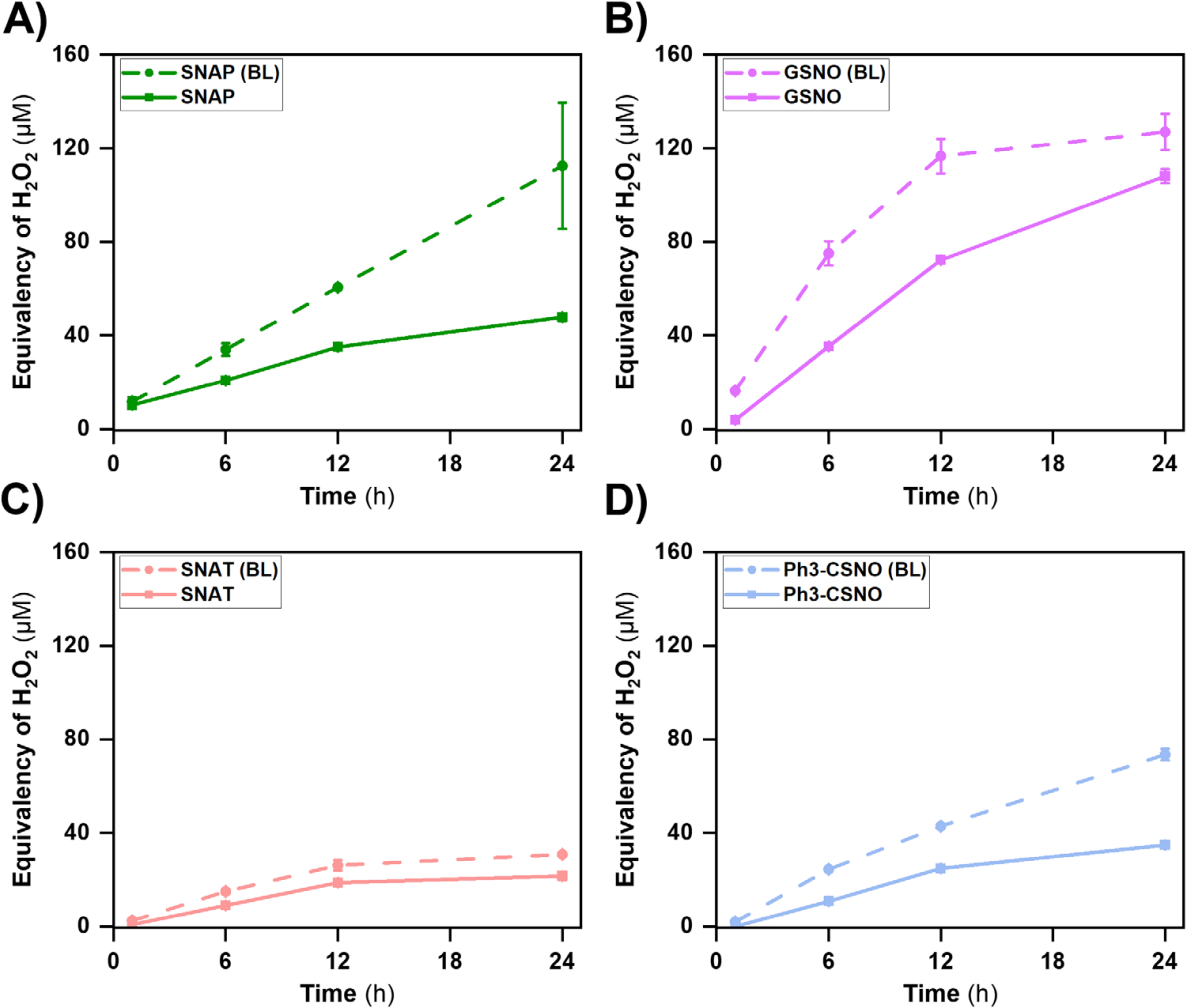
Average equivalency of hydrogen peroxide generation of ROS from RSNO‐coated CIs over 24 h. Increase in ROS production with aBL irradiation from SNAP‐ (A), GSNO‐ (B), SNAT‐ (C), and Ph_3_‐CSNO‐coated CIs (D) under physiological conditions over 24 h. Antimicrobial blue light samples were irradiated throughout the study. Data are shown as mean ± SD (*n* ≥ 3).

The formation of hydrogen peroxide equivalents was evaluated using the Pierce H_2_O_2_ assay to examine oxygen‐centered ROS generation. It should be noted that this assay reports H_2_O_2_‐equivalent oxidative species and may reflect contributions from other reactive species generated in RSNO systems under aBL irradiation. Similar to nitrite, aBL irradiation significantly increased H_2_O_2_ equivalents production for all RSNO‐coated CIs compared to controls (dark conditions) for each individual time point after 6 h. At 24 h, SNAP‐generated H_2_O_2_ equivalents increased from approximately 47.91 ± 1.68 µM under dark conditions to 112.51 ± 26.95 µM with aBL (134.85% increase) (Figure 5A), while GSNO increased from 108.12 ± 2.99 µM to 126.03 ± 7.72 µM (17.49% increase) (Figure 5B). The Ph_3_‐CSNO‐coated CIs exhibited an increase from 34.86 ± 0.91 µM to 73.46 ± 2.42 µM (110.70% increase) (Figure 5C), whereas SNAT demonstrated an enhancement of 42.85%, increasing from 21.57 ± 1.63 µM under dark conditions to 30.82 ± 0.86 µM with aBL (Figure 5D). Although GSNO and SNAP produced higher absolute concentrations of H_2_O_2_, the magnitude of aBL‐induced enhancement was greatest for SNAP and Ph_3_‐CSNO, whereas SNAT exhibited more modest increases relative to its baseline.

These results indicate that aBL does not uniformly amplify RSNO photochemistry, but instead reshapes the balance of primary NO release and secondary redox reactions in a donor‐dependent manner. Absolute nitrite and H_2_O_2_‐equivalent signals followed the trend SNAT < Ph_3_‐CSNO < SNAP < GSNO, reflecting differences in donor loading, hydrophilicity, and aqueous accessibility. However, the extent of aBL‐induced enhancement did not track absolute output, suggesting that photochemical efficiency and secondary oxidation pathways are not governed solely by donor quantity. In particular, SNAT exhibited the largest relative increase in nitrite formation despite lower absolute levels, consistent with a greater sensitivity of its NO‐release pathway to light activation than to bulk reservoir effects. In contrast, SNAP showed the most pronounced increase in H_2_O_2_‐equivalent signal, which may reflect enhanced downstream oxidation chemistry associated with higher local NO flux and greater reactive intermediate formation in solution. These differences indicate that the RSNO structure influences not only total reactive species production but also the efficiency with which light‐driven chemistry propagates into secondary redox pathways. Rather than a uniform ROS amplification, aBL therefore tunes donor‐specific reaction networks, producing distinct reactive environments that may differentially contribute to antimicrobial activity.

### 2.4 Cytotoxicity Evaluation

**FIGURE 6:**
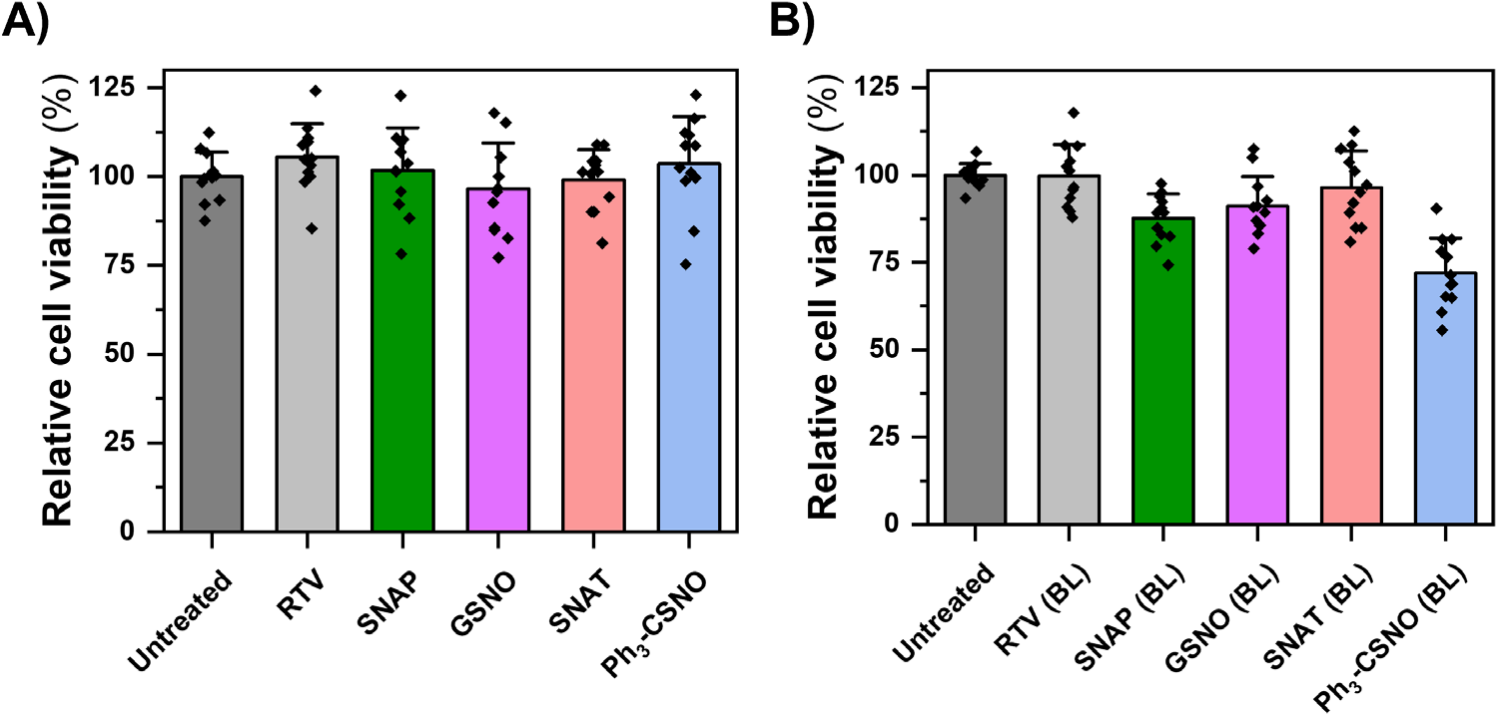
Cytocompatibility analysis of RSNO‐coated CI leachates under (A) dark and (B) aBL‐irradiation conditions against L929 mouse fibroblasts for 24 h. Data are shown as mean ± SD (*n* = 12, 3 biological replicates).

Following evaluation of secondary ROS and RNS generation, the cytocompatibility of the coated CIs was assessed to determine whether the products generated under dark and aBL conditions adversely affect healthy cells. Although NO and ROS play important physiological roles at controlled levels, excessive concentrations may induce cellular stress [30]. Therefore, cytotoxicity studies were performed to confirm that light‐activated RSNO‐coated CIs maintain acceptable biocompatibility under conditions relevant to potential clinical use. Cell viability was evaluated for all RSNO‐coated CIs under both dark (Figure 6A) and aBL conditions (Figure 6B). Across all samples, average cell viability remained above 70%, meeting ISO 10993–5 defined thresholds for cytocompatibility [31].

Irradiation with aBL did not result in a statistically significant reduction in cell viability compared to corresponding dark controls. Although aBL induced greater NO Flux, as well as secondary ROS and nitrite production across all RSNO‐coated CIs, SNAT CIs exhibited the greatest overall cytocompatibility. Exposure to aBL had minimal effects on the cytocompatibility of SNAT and GSNO CIs, with relative cell viabilities remaining comparable between non‐irradiated and irradiated samples (SNAT: 99.09 ± 8.43% vs. 96.49 ± 0.86%; GSNO: 96.63 ± 12.85% vs. 91.13 ± 8.45%). In contrast, aBL irradiation resulted in modest reductions in cell viability for SNAP and Ph_3_‐CSNO CIs (SNAP: 101.84 ± 11.89% vs. 87.69 ± 6.93%; Ph_3_‐CSNO: 103.58 ± 13.29% vs. 71.97 ± 9.99%).

Despite these decreases, all formulations maintained cell viability above the 70% threshold commonly used to define non‐cytotoxic materials. These slight reductions may be attributed to increased local production of NO and ROS under aBL exposure; however, they did not indicate toxic behavior. These results demonstrate that light‐activated RSNO‐coated CIs retain cytocompatibility while generating enhanced levels of reactive species.

### 2.5 Antimicrobial Activity

**FIGURE 7:**
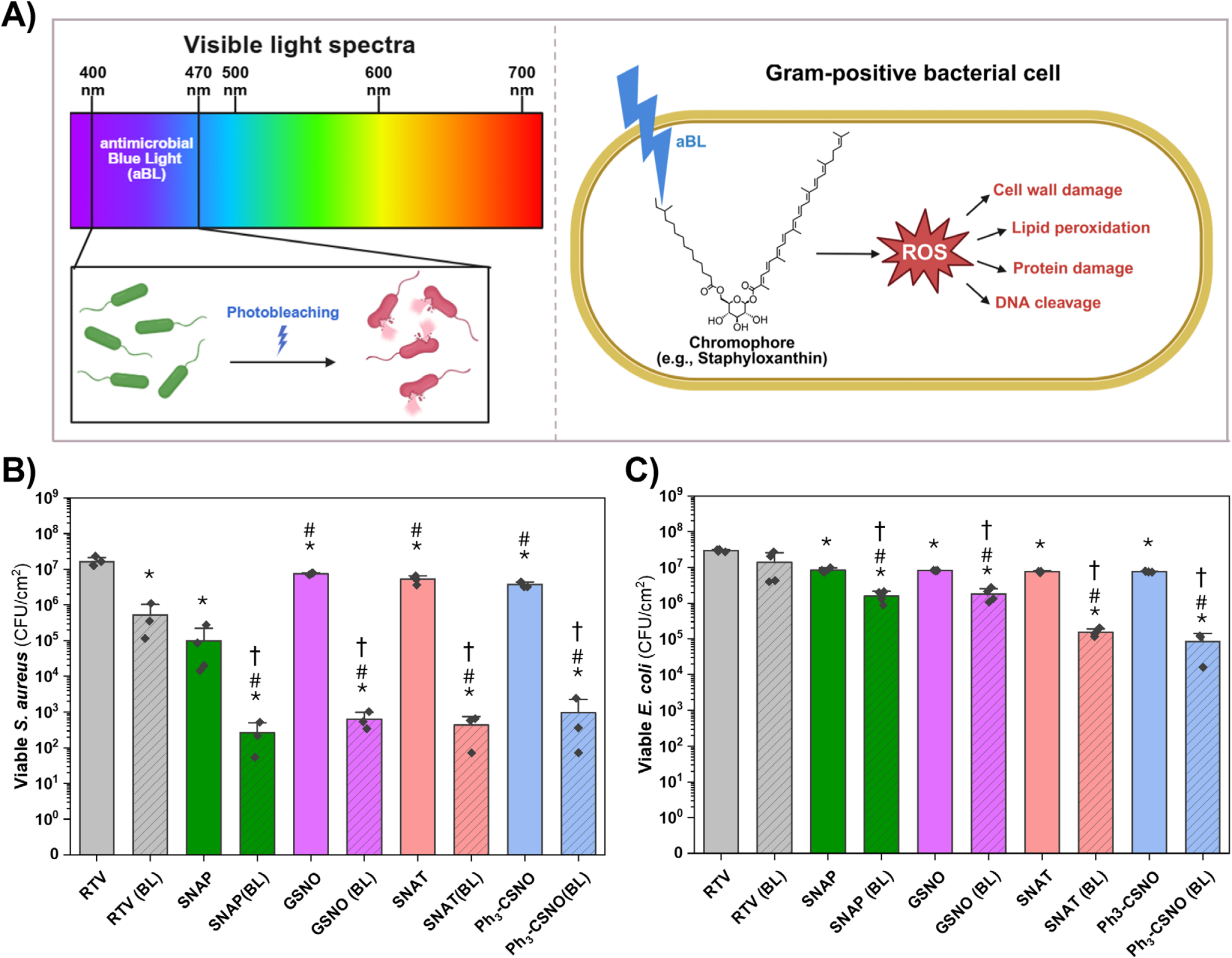
Antibacterial efficacy against planktonic pathogens. (A) Schematic of aBL mechanism of action against representative Gram‐positive bacteria, *S. aureus*. Reduction of viable *S. aureus* (B) and *E. coli* (C) for RTV, SNAP, GSNO, SNAT, and Ph_3_‐CSNO CIs when irradiated with aBL (BL) compared to no light irradiation. Data are shown as mean ± SD (*n* ≥ 3). Symbols signify *p* < 0.05 when comparing all sample types together: ^*^ compared to RTV, ^#^ compared to RTV (BL), ^†^ compared to respective RSNO controls.

Given aBL's ability to inactivate bacterial pathogens, the antibacterial efficacy of the fabricated catheter inserts was evaluated against both Gram‐positive and Gram‐negative bacterial species. *S. aureus* and *E. coli* were selected as representative hospital‐acquired pathogens frequently associated with catheter‐related infections. Previous studies have demonstrated that aBL can significantly reduce *S. aureus* viability by photobleaching the membrane‐associated pigment staphyloxanthin (STX), thereby inducing oxidative stress and compromising membrane integrity (Figure 7A) [32]. This membrane‐associated pigment STX was present in the UV‐vis spectrum after methanol extraction was performed (Figure S8).

When accompanied by aBL, RTV (control) samples were capable of reducing viable planktonic *S. aureus* by more than 1‐log (Figure 7B). However, Dong et al. [32] suggested that photobleaching induced by aBL may only transiently damage *S. aureus*, allowing the bacteria to recover and regrow once irradiation stops and fresh medium is supplied. Therefore, it is necessary to introduce oxidative or nitrosative stress, such as ROS, in combination with aBL to enhance the antibacterial potential [33, 34]. By combining ROS with aBL, light exposure accelerates ROS penetration and uptake through weakened bacterial membranes [35]. Similar to hydrogen peroxide, NO and its secondary reactive species enhanced the reduction of viable planktonic *S. aureus*, achieving up to a 4‐log reduction for all RSNO CIs when combined with aBL irradiation (Figure 7B).

In the absence of aBL, differences in antibacterial activity were observed among the RSNO formulations. The SNAP and GSNO CIs exhibited greater NO release, as well as higher total nitrite and hydrogen peroxide equivalents production within the first 24 h compared with SNAT and Ph_3_‐CSNO CIs. Consistent with this release profile, SNAP CIs produced the greatest reduction in viable *S. aureus* (99.38%, ∼2‐log reduction), exceeding GSNO (53.53%), SNAT (67.32%), and Ph_3_‐CSNO (76.75%) CIs. The SNAP CIs also generated 122.06% and 37.42% more hydrogen peroxide equivalents and accumulated 733.75% and 90.60% more total nitrite than SNAT and Ph_3_‐CSNO CIs, respectively, supporting a relationship between increased reactive species generation and enhanced antibacterial efficacy. Although GSNO CIs generated higher total hydrogen peroxide equivalents and nitrite levels than SNAP CIs within the first 24 h, SNAP CIs exhibited greater antibacterial efficacy. This discrepancy may be attributed to the rapid depletion of the GSNO NO reservoir, which limited sustained NO delivery. In contrast, the greater leaching observed for SNAP (109.10% higher than GSNO) may have increased NO availability throughout the planktonic solution, thereby enhancing bacterial killing. Despite these differences under dark conditions, the addition of aBL eliminated substantial differences between RSNO formulations, with all RSNO‐coated CIs producing comparable (>4‐log) reductions in *S. aureus* viability. These findings indicate that light‐triggered NO release provides robust antibacterial activity regardless of the specific RSNO donor while allowing donor selection to be guided by other material properties, such as stability and release characteristics.

To further evaluate activity against clinically relevant resistant pathogens, the antibacterial efficacy of the fabricated CIs was also assessed against methicillin‐resistant *Staphylococcus aureus* (MRSA) (Figure S9A). Similar trends to those observed for *S. aureus* were obtained, with RSNO‐coated CIs combined with aBL producing the greatest reductions in bacterial viability. Interestingly, aBL alone resulted in greater reductions in MRSA viability than in *S. aureus*. Previous studies have reported that aBL remains highly effective against MRSA due to the presence of endogenous photosensitizers and the retention of staphyloxanthin‐mediated photoinactivation pathways despite antibiotic resistance [36]. Collectively, these findings demonstrate that the antibacterial effects of the RSNO‐coated CIs are maintained against antibiotic‐resistant *S. aureus* strains.

The Gram‐negative bacterium *E. coli* was less susceptible to aBL, resulting in a 52.31% reduction in viable planktonic bacteria (Figure 7C). This reduced susceptibility is attributed to structural differences in Gram‐negative bacteria, including the presence of an additional outer membrane that limits the penetration of antimicrobial agents [37]. In addition, Gram‐negative bacteria generally exhibit lower uptake of small molecules, such as antibiotics [38]. Therefore, RSNO‐coated CIs in the absence of aBL irradiation produced moderate reductions in viable *E. coli*, with comparable antibacterial effects across formulations (SNAP: 71.04%, GSNO: 72.41%, SNAT: 74.51%, and Ph_3_‐CSNO: 74.13%). The addition of aBL irradiation substantially increased bacterial killing across all RSNO‐coated CIs, resulting in reductions of at least 1.3 log. Although SNAT and Ph_3_‐CSNO CIs achieved approximately 2‐log reductions in viable *E. coli*, compared with 1.3‐ to 1.4‐log reductions for SNAP and GSNO CIs, all formulations demonstrated enhanced antibacterial activity following light activation. These species‐dependent differences likely reflect variations in bacterial cell envelope structure and susceptibility to reactive nitrogen and oxygen species rather than differences in the overall effectiveness of light‐triggered NO release.

In addition to bacterial pathogens, the antifungal activity of the fabricated CIs was evaluated against *Candida albicans* (*C. albicans*) (Figure S9B). Unlike the bacterial species examined, aBL irradiation alone resulted in complete elimination of viable fungal cells, consistent with previous reports demonstrating the susceptibility of *C. albicans* to aBL through excitation of endogenous porphyrins and flavins, leading to substantial intracellular ROS generation [9]. Consequently, complete fungal killing was also observed for all RSNO‐coated CIs when combined with aBL irradiation. In contrast, RSNO‐coated CIs in the absence of aBL produced no significant reductions in fungal viability, indicating that aBL was the dominant contributor to antifungal activity under these conditions. These findings demonstrate that combining RSNO‐coated CIs with aBL markedly enhances activity against both Gram‐positive and Gram‐negative bacteria, while aBL alone provides potent antifungal activity against *C. albicans*. Collectively, the fabricated platform exhibits broad‐spectrum antimicrobial efficacy against susceptible bacterial and fungal pathogens.

### 2.6 In Vitro Hemocompatibility Assessment

The strong antimicrobial performance of SNAT and Ph_3_‐CSNO CIs, particularly when combined with aBL irradiation, identified these formulations as the most promising candidates for further evaluation. Prior to in vivo assessment, the hemocompatibility of these blood‐contacting materials must be established. Therefore, hemolysis and platelet adhesion studies were performed to evaluate the blood compatibility of SNAT and Ph_3_‐CSNO CIs with and without aBL irradiation.

Hemolysis testing revealed no detectable erythrocyte damage for any of the evaluated conditions, with all samples exhibiting a hemolytic index of 0 (Table 2).

**TABLE 2:** Hemolytic index of CIs (*n* = 8, 2 biological replicates) following blood exposure.

Hemolytic Index	RTV (Control)	SNAT	Ph_3_‐CSNO
Without BL	0	0	0
With BL	0	0	0

These findings demonstrate that neither the RSNO coatings nor aBL irradiation adversely affect red blood cell integrity during blood exposure. The complete absence of hemolysis further supports the suitability of the fabricated CIs for blood‐contacting applications and suggests that the incorporation of RSNO donors and exposure to aBL do not compromise erythrocyte compatibility.

**FIGURE 8:**
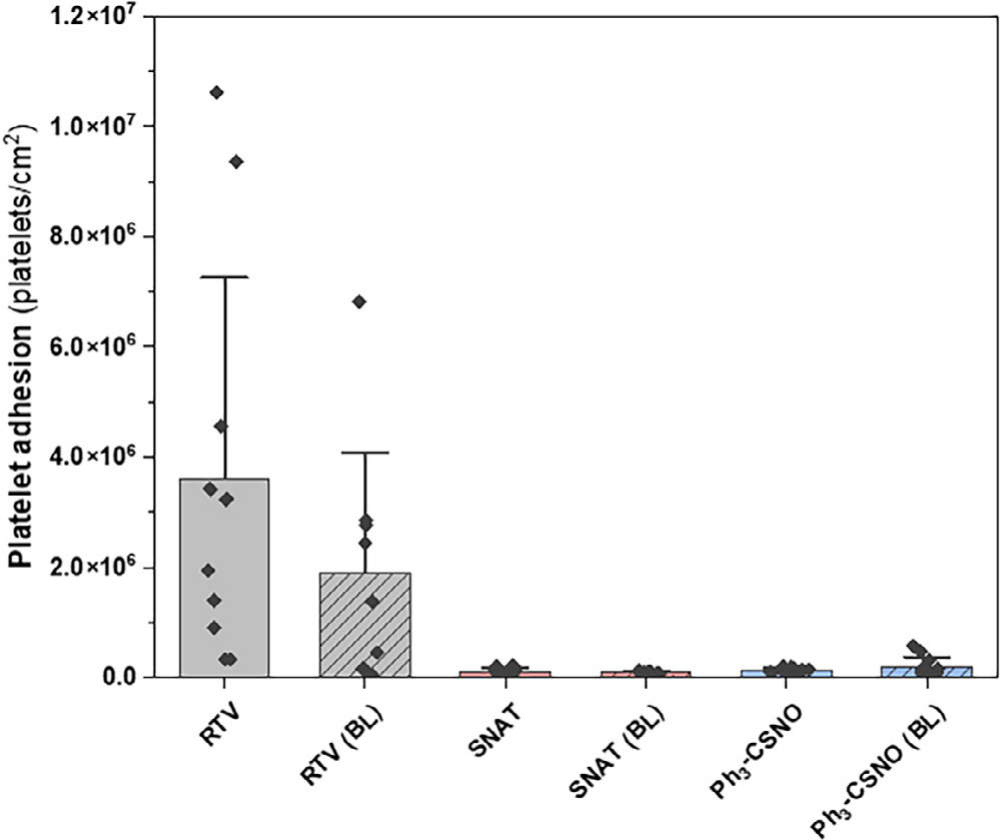
Platelet adhesion on control (RTV), SNAT, and Ph_3_‐CSNO CIs with and without aBL (BL) irradiation, quantified by LDH assay. Data are shown as mean ± SD (*n* ≥ 10, 3 biological replicates). ^*^ signifies *p* < 0.05 compared to RTV.

Platelet adhesion was quantified using an LDH assay. Exposure to aBL alone resulted in a modest but non‐significant reduction in platelet adhesion relative to the control surface (Figure 8). Previous in vitro studies have reported that low‐level aBL irradiation can reduce platelet adhesion under certain shear conditions, suggesting that platelet responses to light may depend on wavelength, dose, and hemodynamic environment [39]. In addition, both SNAT and Ph_3_‐CSNO CIs significantly reduced platelet adhesion in the presence and absence of aBL irradiation, with SNAT and Ph_3_‐CSNO reducing adhesion by approximately 97% and 95%, respectively.

These reductions are consistent with the well‐established antiplatelet properties of NO, which inhibits platelet activation, aggregation, and surface adhesion through cyclic guanosine monophosphate (cGMP)‐mediated signaling pathways [40]. Notably, no significant differences were observed between SNAT and Ph_3_‐CSNO CIs, suggesting that both formulations provided comparable antithrombotic performance.

Although both SNAT and Ph_3_‐CSNO CIs demonstrated favorable hemocompatibility and antimicrobial activity, SNAT was selected for subsequent in vivo evaluation. While the antibacterial performance of the two formulations was comparable, SNAT maintained greater cytocompatibility following aBL irradiation. In contrast, the viability of cells exposed to Ph_3_‐CSNO CIs approached the accepted threshold for non‐cytotoxic materials. Therefore, SNAT was identified as the lead candidate for animal studies due to its combination of antimicrobial efficacy, hemocompatibility, and enhanced cytocompatibility.

### 2.7 In Vivo Antibacterial Evaluation of SNAT CIs via 7 h Rabbit Model

**FIGURE 9:**
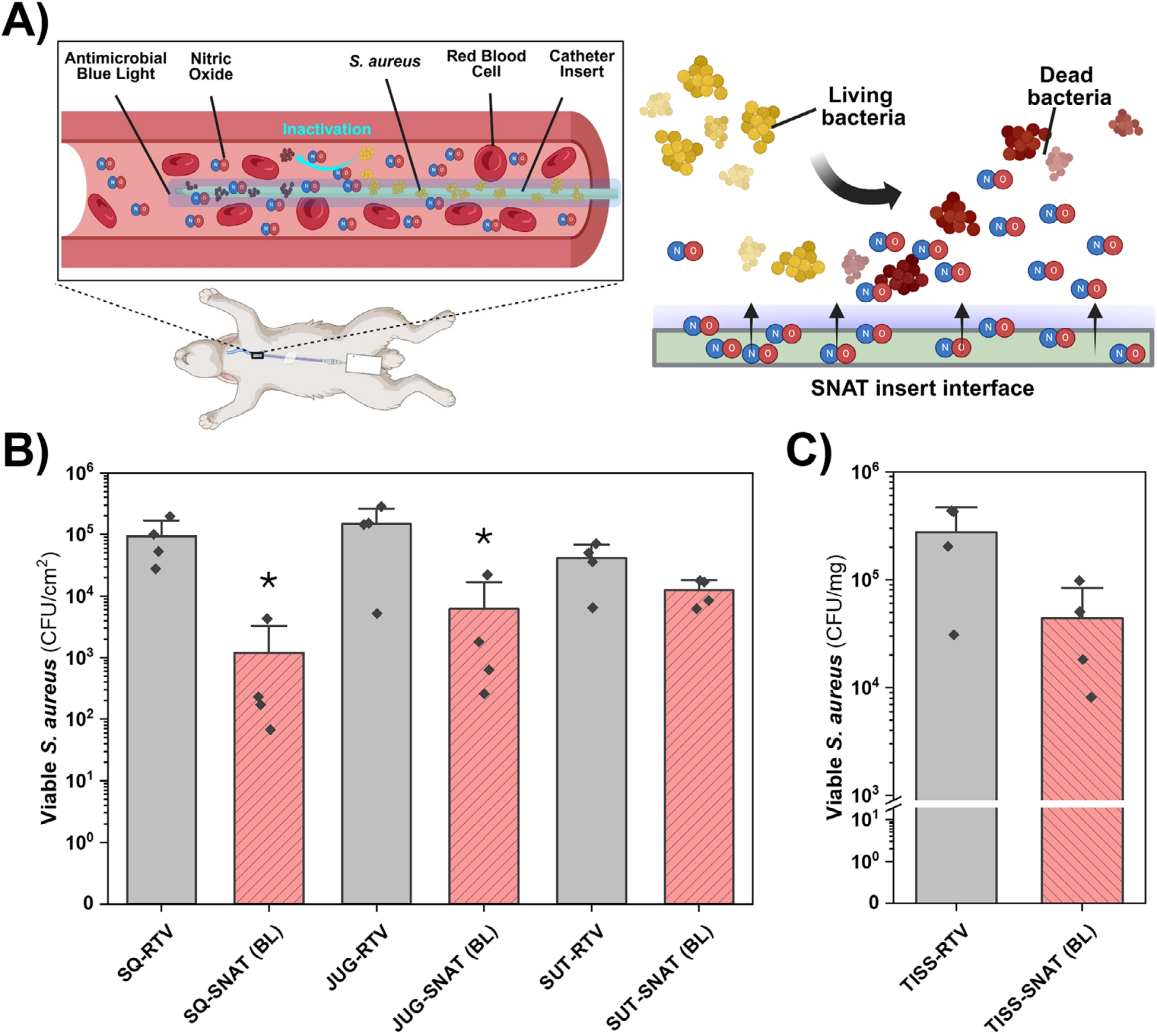
In vivo evaluation of SNAT‐coated CIs combined with aBL (BL) in a 7 h rabbit catheter infection model. (A) Schematic representation of catheter‐associated bacterial colonization and inhibition through NO release and aBL‐mediated antibacterial activity. (B) Viable *S. aureus* recovered from catheter regions including subcutaneous (SQ), jugular (JUG), and suture (SUT) sites, normalized to surface area (CFU/cm^2^). (C) Bacterial burden recovered from surrounding tissue (CFU/mg). Data are shown as mean ± SD (*n* = 4). ^*^ signifies *p* < 0.05 compared to respective controls.

The ability of SNAT‐coated CIs, combined with aBL irradiation, to reduce bacterial burden in vivo was evaluated using a rabbit catheter infection model (Figure 9A). Catheter‐associated infections remain a significant clinical challenge, as contamination of indwelling devices can rapidly lead to surface colonization, biofilm formation, and dissemination of pathogens into surrounding tissues [41]. Therefore, demonstrating antimicrobial efficacy under physiologically relevant conditions is critical for assessing the translational potential of infection‐resistant catheter technologies. To evaluate spatial bacterial persistence along the implanted device, viable *S. aureus* recovered from the subcutaneous (SQ), jugular (JUG), and suture (SUT) regions of the catheter insert, as well as surrounding tissue (TISS), was quantified following 7 h of implantation. Relative to RTV control catheter inserts without aBL irradiation, SNAT‐coated CIs with aBL exposure reduced bacterial burdens across all evaluated regions. Significant decreases were observed in the subcutaneous and jugular segments, with reductions of 2.35‐ and 1.68‐logs, respectively (Figure 9B). More modest reductions were observed at the suture interface (0.43‐log) and in surrounding tissue (0.80‐log) (Figure 9B,C).

The pronounced reductions in the subcutaneous and jugular regions are particularly relevant, as these interfaces are highly susceptible to early‐stage bacterial colonization during implantation. In particular, the jugular segment is in direct contact with the bloodstream and may serve as a conduit for systemic dissemination [42]. Limiting bacterial attachment at these early interfaces is critical, as initial adhesion events strongly influence subsequent biofilm development and long‐term device‐associated infection [43]. The observed decrease in bacterial counts on the surrounding tissue samples suggests that antimicrobial activity may extend beyond the catheter surface. This trend is consistent with the diffusion of nitric oxide and reactive intermediates into adjacent microenvironments, potentially limiting local bacterial survival [44]. These in vivo results are consistent with the in vitro findings and demonstrate effective suppression of early‐stage bacterial colonization across clinically relevant catheter interfaces.

## 3 Discussion

Light‐activated NO release from RSNO‐functionalized catheter inserts is governed by contributions from donor chemistry and irradiation wavelength, which together define NO flux, release duration, and reactive species generation [25, 45]. Structural differences among RSNO donors influence both stability and decomposition pathways, while light activation provides external control over S─N bond cleavage and downstream NO production. Donor accessibility and polymer confinement further modulate photochemical efficiency under irradiation, enabling tunable generation of NO and secondary reactive nitrogen and oxygen species under aBL [46]. These findings are consistent with prior reports that RSNO‐derived NO can participate in downstream redox cycling reactions, particularly in oxygen‐rich microenvironments under photo‐stimulation [47]. More stable or hydrophobically constrained RSNO environments support prolonged NO release by limiting premature loss and enhancing photostability, whereas less constrained systems favor rapid initial release with reduced duration of activity [48, 49].

An additional consideration may be the fate of the donor following NO release. Since SNAT and Ph_3_‐CSNO remain retained within the catheter insert rather than diffusing into the surrounding environment, subsequent donor decomposition products are likewise expected to remain localized within the polymer matrix. In contrast, leachable RSNOs such as SNAP or GSNO may release both NO and their corresponding thiol products into the surrounding medium, where subsequent oxidation can lead to disulfide formation [46]. Although these downstream products were not directly quantified in the present study, their potential contribution to the antimicrobial and cytocompatibility responses cannot be completely excluded. Further characterization of RSNO photolysis products would therefore be necessary to fully establish their contribution to the biological effects observed.

The previously reported and proposed antibacterial effect of aBL against *Staphylococci*, such as *S. aureus*, is based on the absorption of aBL by endogenously available STX, which decreases membrane integrity [50]. The availability of STX and its biosynthesis precursors in *S. aureus* was confirmed via STX extraction and subsequent UV‐vis spectroscopy, resulting in a similar extraction ratio between the precursor 4,4′‐diapophytoene and STX (7:1) (Figure S8) [51]. Hence, the presence of STX was confirmed, as well as a ∼1‐log reduction with RTV‐coated CIs and aBL treatment. The antibacterial action of aBL due to STX absorption could be further confirmed. As a result of the effect of aBL on the membrane integrity of *Staphylococci*, the addition of oxidative stress via nitric oxide release significantly increased the antibacterial activity.

The previously discussed advantages of RSNO‐coated CIs translated into significant antimicrobial activity, with up to ∼4‐log reductions under combined NO and aBL treatment. This level of bacterial reduction exceeds many passive antimicrobial coatings and compares favorably with previously reported NO‐releasing or light‐activated systems, which typically achieve 1–3 log reductions depending on material composition and exposure conditions [14, 15]. Achieving approximately a four‐order‐of‐magnitude reduction is particularly significant for catheter‐associated infections, where relatively small populations of adherent bacteria can rapidly proliferate into mature biofilms that are substantially more tolerant to antimicrobial agents. Consequently, suppressing bacterial adhesion and early colonization has a greater impact on preventing device‐associated infections than attempting to eradicate established biofilms. Therefore, smartphone‐guided RSNO‐coated CIs may provide a noninvasive strategy for localized disinfection of existing catheters, such as peripheral intravenous catheters, by inserting the CI into the catheter lumen prior to blood sampling or intravenous fluid infusion. This compatibility with existing catheter technologies could potentially extend catheter dwell time and reduce the need for repeated catheter replacement, thereby minimizing additional catheterization procedures and associated patient burden [52]. Moreover, the synergistic effect of NO and aBL likely arises from concurrent membrane disruption, metabolic inhibition, and oxidative stress, creating a hostile microenvironment that limits bacterial survival and early biofilm establishment. This antibacterial efficacy is achieved without compromising mammalian cell or blood compatibility. The modest reduction in cell viability observed for Ph_3_‐CSNO, while remaining within accepted cytocompatibility criteria, likely reflects the greater production of reactive species under aBL irradiation and illustrates the trade‐off between maximizing antimicrobial potency and preserving host compatibility when selecting RSNO donors. Hemocompatibility assessments demonstrated minimal hemolysis and platelet activation, indicating that localized NO delivery remained within a therapeutic window capable of suppressing bacterial colonization without inducing subsequent blood toxicity. Maintaining this balance is particularly important as excessive NO flux or uncontrolled reactive nitrogen and oxygen species generation can promote oxidative damage to mammalian cells and blood components [53]. The favorable hemocompatibility observed here highlights the ability of controlled, light‐activated NO release to maximize antimicrobial efficacy while minimizing adverse host responses, representing an important challenge in the development of antimicrobial blood‐contacting devices. This balance is especially critical for catheter applications, where materials remain in continuous contact with blood while simultaneously resisting bacterial adhesion and biofilm formation. Consistent with these in vitro findings, SNAT‐coated catheter inserts also significantly reduced bacterial colonization in vivo, demonstrating that the combined optimization of donor chemistry, light‐triggered NO release, and hemocompatibility translates to effective antimicrobial performance under physiologically relevant conditions. Therefore, SNAT‐coated CIs represent a promising antibiotic‐free antibacterial alternative for blood‐contacting catheters, such as central venous catheters. Furthermore, the ability to externally activate NO release provides an advantage over conventional passive coatings by enabling antimicrobial therapy to be delivered only when needed, thereby reducing unnecessary donor consumption while permitting repeated treatment throughout catheter residence. Another advantage is the ease of use and the replaceability of CIs. Hence, the CI can be noninvasively replaced unlike actively releasing antibiotic‐coated or impregnated central venous tunneled catheters before the depletion of the nitric oxide payload, providing continuous antibacterial properties during treatment.

Despite these promising outcomes, several limitations warrant consideration. The current system relies on external light delivery, which may limit activation in deeply implanted or poorly accessible sites. The in vivo study did not include separate SNAT‐only or aBL‐only treatment groups, limiting the ability to independently quantify their contributions to the observed antibacterial activity. However, preliminary in vitro studies demonstrated that both SNAT and aBL independently reduced viable adhered *S. aureus* by 0.87 and 0.81 log units, respectively, while the combined SNAT and aBL treatment produced a 1.52‐log reduction (Figure S10). Additionally, the 7‐h rabbit model was designed to evaluate the ability of the CIs to prevent or reduce initial bacterial colonization rather than eradicate established biofilms. While this short‐duration model demonstrated reduced bacterial burden under physiologically relevant conditions, longer‐term studies will be necessary to evaluate the efficacy of the CIs against mature biofilms and established catheter‐associated infections. Mature biofilms are inherently more tolerant to reactive nitrogen species and oxidative stress due to their extracellular polymeric matrix and altered metabolic state. Oxygen availability may further influence the relative contribution of aBL‐mediated ROS generation, as oxygen diffusion can be limited within mature biofilms and potentially reduce the efficacy of oxygen‐dependent photochemical processes. However, NO and its reactive derivatives have been reported to promote biofilm dispersal and exhibit direct antibiofilm activity, which may disrupt the biofilm environment and improve oxygen and reactive species access to bacterial cells. Thus, NO‐mediated antimicrobial activity may provide complementary effects that are less dependent on the oxygen availability required for aBL‐mediated ROS generation. Future studies under controlled hypoxic conditions will be necessary to directly evaluate the contribution of oxygen availability to the combined aBL and NO antimicrobial mechanism. Furthermore, the connection between light source and catheter insert required for light activation necessitated continuous anesthesia and monitoring of the animals, which limited the practical duration of the present in vivo study [54]. Future studies may explore improved light delivery strategies, including the incorporation of phosphorescent or persistent luminescent materials capable of extending local optical stimulation following a single activation event, thereby reducing the need for continuous external irradiation [55].

## 4 Conclusions

The combination of smartphone‐guided antimicrobial blue‐light irradiation and nitric oxide release represents a powerful, antibiotic‐independent approach for combating device‐associated infection. In vitro studies demonstrated that aBL combined with RSNO‐coated CIs produced substantial reductions in bacterial viability, including up to a 4‐log reduction in *Staphylococcus aureus* viability, while also maintaining cytocompatibility across all formulations. All RSNO CIs exhibited controllable, wavelength‐dependent NO release upon irradiation, supporting the use of light as an external trigger for spatiotemporally regulated antimicrobial therapy. The in vitro hemocompatibility assessments further confirmed that selected formulations were non‐hemolytic and significantly reduced platelet adhesion, indicating suitability for blood‐contacting applications. Based on the combined antimicrobial performance and cytocompatibility profile, SNAT and Ph_3_‐CSNO emerged as the lead candidates for further evaluation, with SNAT ultimately selected due to its improved overall safety margin. In a clinically relevant 7‐h rabbit catheter infection model, SNAT‐coated CIs combined with aBL significantly reduced bacterial burden across all catheter regions, including subcutaneous, jugular, and suture interfaces, as well as surrounding tissue, compared with control devices. These results demonstrate that phototriggered NO release can effectively suppress bacterial colonization and limit early infection dissemination along implanted medical devices in vivo.

This work establishes a light‐responsive NO‐releasing catheter platform that integrates complementary oxidative and nitrosative antimicrobial mechanisms while preserving blood and cellular compatibility. By coupling aBL with on‐demand NO delivery, this strategy overcomes key limitations of conventional antimicrobial coatings and provides a versatile framework for combating antimicrobial resistance at the device–tissue interface. The SNAT‐based platform is particularly promising for intravascular device applications and may be broadly adaptable to other indwelling medical devices susceptible to biofilm formation and infection.

## 5 Materials and Methods

### 5.1 Materials

DOWSIL 3140 MIL‐A‐46146 RTV silicone rubber (SR) was purchased from Ellsworth Adhesive (Germantown, WI), DOW Corporation, USA. Multi‐Color Smart Modules with Fibrance Light‐Diffusing Fiber and fiber reels were purchased from Versalume (Santa Clara, CA). Concentrated hydrochloric acid (conc. HCl, 12.1 M), and ethanol (100%) were purchased from Fisher Scientific, Inc. Luria‐Bertani (LB) broth, LB agar, phosphate buffered saline (PBS), sodium nitrite (>99.0%), methanol (>99.8%), concentrated sulfuric acid (conc. H_2_SO_4_, 18 M), ethylenediaminetetraacetic acid (EDTA), *N*‐acetylpenicillamine (NAP), triphenylmethanethiol (>97%), and tetrahydrofuran (THF) were purchased from Sigma‐Aldrich (St. Louis, MO). _L_‐Glutathione (reduced >98%) was obtained from Alfa Aesar (Ward Hill, MA). 1‐adamantanethiol (NAT, >95%) was purchased from Oakwood Chemicals (Estill, SC), and Calcium‐ and Magnesium‐Free Phosphate Buffered Saline (CMF‐PBS, Corning) was purchased from VWR. All other materials were purchased from Fisher Scientific, Sigma‐Aldrich, or VWR. Phosphate‐buffered saline (10 mM, pH 7.4) and LB media were dissolved in deionized (DI) water and sterilized in an autoclave at 121°C, 100 kPa (15 psi) above atmospheric pressure for 30 min prior to bacterial studies. *Escherichia coli* (ATCC 25922), *Staphylococcus aureus* (ATCC 6538), methicillin‐resistant *Staphylococcus aureus* (ATCC BAA 41), and *Candida albicans* (ATCC MYA4441) were obtained from the American Type Culture Collection (Manassas, VA).

### 5.2 Synthesis of RSNOs

#### 5.2.1 *S*‐Nitroso‐*N‐*acetylpenicillamine (SNAP) Synthesis

Synthesis of SNAP was performed using a slightly modified version of a previously reported method, yielding crystals with over 90% purity [13]. Briefly, 5 g of sodium nitrite was dissolved in 40 mL of deionized (DI) water and added dropwise to a stirring solution containing 5 g of NAP in 60 mL of methanol, 5 mL of concentrated H_2_SO_4_, and 20 mL of concentrated HCl. The reaction vessel was placed in an ice bath and continuously purged with nitrogen for 8 h without stirring. Crystals formed were collected via vacuum filtration, gently rinsed with 100 µL of chilled deionized water (DI) water, and dried overnight in a desiccator. All steps were conducted under light‐protected conditions. Crystal purity was assessed using a chemiluminescence Nitric Oxide Analyzer (NOA 280i, Sievers, Boulder, CO) by quantifying the amount of NO released from a known concentration of SNAP in the presence of 50 mM copper chloride and 10 mM cysteine.

#### 5.2.2 *S*‐Nitrosoglutathione (GSNO) Synthesis

Synthesis of GSNO was performed as previously described [56]. Briefly, 5 g of reduced glutathione was dissolved in a mixture of concentrated HCl and DI water, and subsequently cooled in an ice bath. An excess of sodium nitrite (1.2 g) was added, and the reaction mixture was kept in the ice bath for 40 min. Chilled acetone was introduced to precipitate GSNO, which was collected via vacuum filtration and washed with additional chilled acetone and DI water. The resulting GSNO was dried overnight under vacuum and stored in the dark at −20°C until further use. Purity was assessed using a NOA by quantifying the amount of NO released from a known concentration of GSNO in the presence of 50 mM copper chloride and 10 mM cysteine.

#### 5.2.3 *S*‐Nitroso‐1‐adamantanethiol (SNAT) Synthesis

Synthesis of SNAT was performed according to a previously reported method with slight modifications [57]. Briefly, 5 g of 1‐adamantanethiol was added to an amber 500 mL round‐bottom flask (RBF), followed by 100 mL of chloroform and 80 mL of 0.1 M HCl. Sodium nitrite (2.5 g) was introduced, and the flask was placed on a shaker at 250 rpm for 1 h. The reaction mixture was purified by separating the organic (chloroform) and aqueous layers using a separatory funnel. The organic phase was washed twice with 80 mL of DI water. After purification, the chloroform was removed using a rotary evaporator, yielding SNAT as a dry powder, which was stored at −20°C until use.

#### 5.2.4 *S*‐Nitrosotriphenylmethanethiol (Ph_3_‐CSNO) Synthesis

Synthesis of Ph_3_‐CSNO was performed according to a previously reported method with slight modifications [58]. Briefly, 5 g of triphenylmethanethiol was added to an amber 500 mL RBF, followed by 100 mL of chloroform and 80 mL of 0.1 M HCl. Sodium nitrite (2.5 g) was introduced, and the flask was placed on a shaker at 250 rpm for 1 h. The reaction mixture was purified by separating the organic (chloroform) and aqueous layers using a separatory funnel. The organic phase was washed twice with 80 mL of DI water. Following purification, chloroform was removed via rotary evaporation, yielding Ph_3_‐CSNO as a dry powder. The final product was stored at −20°C until use.

### 5.3 Prediction of Octanol‐Water Partition Coefficients

The octanol‐water partition coefficient *P* (log*P*) was predicted for all RSNO with the ChemDraw software (PerkinElmer, Waltham, PA, USA), which is based on the Wildman‐Crippen method [59, 60]. The hydrophilicity and lipophilicity of the RSNOs were determined according to the scale reported by Cumming and Ruecker's [61]: very hydrophilic (log*P* = −3) and extremely hydrophobic (log*P* = 10).

### 5.4 NO‐Releasing Catheter Insert (CI) Fabrication

Side‐glow Fibrance Light‐Diffusing Fiber (FLDF) consisted of an internal silica glass core, an acrylate polymer cladding, and a poly(vinyl chloride) (PVC) jacket (outer diameter (OD): 0.9 mM). For dip‐coating, Fibrance fibers were cut into 6.5 cm segments, with the internal core slightly extended beyond the bottom of each segment to avoid coating the interior of the PVC jacket.

Nitric oxide‐releasing catheter inserts (CIs) were fabricated by dip‐coating the PVC jacket with RSNO‐incorporated RTV silicone. First, 800 mg of RTV silicone was dissolved in 4 mL of THF in a 20 mL scintillation vial at room temperature. In parallel, 20 wt% RSNO was dissolved in 4 mL of THF in a separate vial until homogeneous. The RTV solution was added to the RSNO solution to create a 100 mg/mL RTV mixture. Using an amber 4 mL vial, each fiber segment was dip‐coated 20 times, with 1‐min drying intervals between coats and fiber rotation every 5 coats to ensure uniform coverage. Samples were left to dry overnight in a fume hood at room temperature, protected from light.

After drying, the coated segments were trimmed to 3 cm by removing the bottom end, where the coating may have accumulated due to gravity. Each 3 cm RSNO‐coated segment was then mounted onto a new up to 20 cm fiber core, which was attached to an LC connector for use with the Multi‐Color Smart Modules. To secure the RSNO segment to the longer fiber core, two additional dip‐coats of 100 mg/mL RTV were applied using the same method. Final assemblies were dried overnight in the fume hood and stored at −20 °C until use.

### 5.5 Scanning Electron Microscopy and Energy‐Dispersive X‐ray Spectroscopy

Surface characterization of the catheter inserts (CIs) was performed using microscopy and spectroscopy techniques to evaluate their morphology and elemental composition. Samples were sputter‐coated with a 10 nm gold–palladium layer using a Leica sputter coater (Leica Microsystems) prior to imaging. A variable‐pressure scanning electron microscope (Hitachi SU3900) was used to examine the surface and cross‐sectional morphology of the different CIs. Energy‐dispersive X‐ray spectroscopy (EDS) was performed to determine the presence and distribution of nitrogen and sulfur within the RSNO‐containing sponges.

### 5.6 Optical and Material Properties of the Multi‐Color Smart Modules (MCSM)

The MCSM in combination with the Fibrance Light‐Diffusing fiber is capable of irradiating red (λ = 639 nm, power output < 20 mW), green (λ = 515 nm, power output < 10 mW), and blue (λ = 450 nm, power output < 20 mW) light with a numerical aperture of >0.5. The Fibrance Light‐Diffusing Fiber consists of a pure silica glass core (OD: 170 ± 3 µm) and an acrylate polymer cladding (OD: 230 ± 10 µm), which is surrounded by a loose optical‐grade PVC tube (OD: 900 µm).

A smartphone app (Versalume, Santa Clara, CA, USA) was used to control the smartphone‐guided light irradiation. In brief, when the MCSMs were turned on, a Bluetooth connection between the individual MCSM and the smartphone was established. Based on the experiment, blue, green, or red light intensity was set to 100% via the application (Figure S11).

### 5.7 Light Emission Detection

To characterize the emission wavelengths of the Multi‐Color Smart Modules, a wireless spectrophotometer (PS‐2600, PASCO Scientific; detection range: 380–950 nm) was used. The Fibrance fiber, without the protective PVC jacket, was positioned securely with its output directed toward the detector, and spectral measurements were taken with an integration time of 10 ms across three light settings: red, green, and blue. The light color was adjusted in real time via a Bluetooth‐connected smartphone application, enabling uninterrupted modulation of the emission. All measurements were performed in a dark environment to eliminate interference from ambient light and ensure accurate characterization of each individual light source.

### 5.8 Photo‐Triggered No Release

Nitric oxide release from RSNO samples, both with light exposure (e.g., SNAP–Blue Light) and without (e.g., SNAP), was quantified using the Zysense chemiluminescence NOA, widely regarded as the gold standard for NO detection. Measurements were conducted under a nitrogen atmosphere and physiological conditions (10 mM PBS with 100 µM EDTA, pH 7.4, at 37°C). The sample chamber was continuously swept with nitrogen at approximately 150 mL min^−1^ while an additional 50 mL min^−1^ nitrogen purge maintained an inert atmosphere, resulting in a total gas flow of approximately 200 mL min^−1^ through the analyzer [62]. The reaction cell pressure was maintained at ∼5.6 Torr and the supply pressure at ∼9.5 psi. The rate of NO release was normalized to the total surface area of the sample, including both the PVC‐coated jacket and internal core, and reported in units of mol min^−1^ cm^−2^.

#### 5.8.1 Wavelength‐Dependent NO Release

The effect of different light colors on NO release from RSNO‐coated CIs was investigated. Prior to NO release assessment, all samples were incubated in 2 mL of 10 mM PBS with 100 µM EDTA at 37°C for 2 h. Preincubation was performed in the dark to achieve accelerated steady‐state conditions. Samples were placed in an amber NOA sample cell containing 7 mL of 10 mM PBS with 100 µM EDTA at 37°C. After establishing a baseline, the CIs were connected to the MCSM positioned outside the closed‐chamber system. Nitric oxide release was first allowed to reach a steady‐state profile under dark conditions. Using the Bluetooth‐connected smartphone application, the light source was activated at 100% intensity, and NO flux was recorded as the emission color was sequentially changed (20 min intervals). Three light colors (red, green, and blue) were evaluated for their impact on NO release.

#### 5.8.2 Physiological NO Release

Continuous NO release under different light colors was evaluated for various RSNO‐coated fiber formulations. Samples were placed in an amber NOA sample cell containing 7 mL of 10 mM PBS with 100 µM EDTA at 37°C, and baseline measurements were recorded. Samples were connected to the MCSM positioned outside the sealed chamber and allowed to reach a steady‐state NO release profile. For light‐activated groups, illumination began immediately upon placement into the sample cell, with red‐, green‐, and blue‐light conditions assessed. Control samples were not illuminated and protected from light influence. Between measurements, all samples were stored fully submerged in 2 mL PBS with EDTA within 2 mL microcentrifuge tubes at 37°C for up to 7 days. Light‐activated samples remained continuously illuminated throughout both testing and storage.

To evaluate whether temperature changes associated with prolonged light irradiation could contribute to RSNO decomposition, the surface temperature of RSNO‐coated CIs was monitored over 24 h using a handheld ShotPRO thermal imaging camera (Seek Thermal). Temperature measurements were recorded under the same light‐irradiation conditions used for NO release experiments.

### 5.9 Nitrite Accumulation

The NO production of the RSNO‐coated CIs in aqueous conditions was further assessed by quantifying the accumulation of nitrite using the Griess assay. Briefly, 3 cm samples were immersed in 2 mL of CMF‐PBS and incubated at 37°C for predetermined time points (6 h, 12 h, and 24 h). At each interval, a 10 µL aliquot was withdrawn and combined with 90 µL of Griess reagent to achieve a final reagent concentration of 20 mg mL^−1^. The resulting azo dye formation was quantified by measuring absorbance at 540 nm using a microplate reader (BioTek Cytation 5). Sodium nitrite calibration standards ranging from 0 to 40 mM were used to generate a standard curve, enabling conversion of absorbance values to nitrite concentration. The molar extinction coefficient at 540 nm was determined to be approximately 880 M^−1^ cm^−1^.

### 5.10 Hydrogen Peroxide Equivalents Production

Hydrogen peroxide production was quantified using the Pierce Quantitative Peroxide Assay. Briefly, 3 cm samples were immersed in 2 mL of CMF‐PBS and incubated at 37°C for predetermined time points (6 h, 12 h, and 24 h). The assay working solution was freshly prepared by combining Reagent A and Reagent B at a 1:100 (v/v) ratio. For analysis, 20 µL of the sample was added to 200 µL of the working solution in a 96‐well plate, and the mixture was allowed to react for 4 h. Blank controls were prepared by replacing the sample with DI water. Absorbance was then measured at 560 nm using a microplate reader.

### 5.11 In Vitro Antimicrobial Assay

#### 5.11.1 Growth of Microbial Cultures

A single isolated colony of *E. coli*, *S. aureus*, and MRSA was inoculated in LB broth and incubated at 37°C for 15 h at 150 rpm. Bacterial growth in culture media over time was tracked by measuring the optical density (OD) of the suspensions at 600 nm using a UV–vis spectrophotometer (Genesys, Thermo Fisher Scientific). Cultures were reinoculated, and cells in the mid‐logarithmic phase were collected by centrifugation at 4400 rpm for 7 minutes. The OD_600_ of the bacterial suspension was standardized to 0.1, corresponding to approximately 10^7^ colony‐forming units (CFU)/mL, for subsequent experiments. Similarly, *C. albicans* was grown following the same procedures using YM broth.

#### 5.11.2 Antimicrobial Activity Against Planktonic Microbes

The antimicrobial activity of RSNO‐coated CIs was assessed against planktonic microbes, which is important as planktonic cells represent the initial stage of infection before biofilm formation [63]. Samples were initially subjected to UV sterilization for 30 min. Subsequently, CIs were incubated for 24 h at 37°C in sterile 2 mL microcentrifuge tubes containing 2 mL of adjusted microbial suspension (OD_600_ 0.1, ∼10^7^ CFU/mL) in sterile 10 mM PBS. Antimicrobial blue‐light samples were continuously illuminated throughout the study. After 24 h, CIs were removed from the tubes, and the microbial suspensions were serially diluted and plated onto the respective LB or YM agar plates using a bacterial spiral plater (Eddy Jet 2, IUL Instruments). The plates were further incubated at 37°C for 24 h to allow colonies to grow for CFU counting. Viable CFUs from the study were enumerated using an automated bacterial colony counter (Sphere Flash, IUL Instruments), and the number of viable CFUs per cm^2^ was determined. The log reduction in viable microbes compared to RTV controls without aBL was calculated using Equation (1).

(1) $$\mathrm{Log}\;\mathrm{reduction}\;\mathrm{in}\;\mathrm{viable}\;\mathrm{microbes}=\log_{10}\left(\frac{{\mathrm{CFU}}_{\mathrm{control}}}{{\mathrm{CFU}}_{\mathrm{treatment}}}\right)$$

### 5.12 In Vitro Cytotoxic Evaluation

Cytotoxicity of the RSNO‐coated side‐glowing fiber optics was assessed using L929 mouse fibroblasts (NCTC clone 929, ATCC). The fibroblasts were cultured in Eagle's Minimum Essential Medium (EMEM, ATCC), supplemented with 10% Fetal Bovine Serum (VWR) and 1% Penicillin‐Streptomycin at 37°C under a humidified atmosphere and 5% CO_2_ / 95% air.

The methylthiazoletetrazolium (MTT) assay was used to evaluate the cytotoxic effects of the aBL‐triggered nitric oxide on L929 fibroblasts. The cytotoxic evaluation was performed in accordance with ISO 10993–5. Fibroblasts were seeded into 96‐well plates and cultured to approximately 70% subconfluency prior to exposure to the sample leachates. The culture medium was then removed, and the cells were treated with leachates of the RSNO‐coated samples with and without aBL for 24 h. The leachates were UV‐sterilized for 30 min and collected in 2 mL of EMEM with 10 mM 2‐[4‐(2‐hydroxyethyl)piperazin‐1‐yl]ethanesulfonic acid (HEPES) buffer for 24 h, with or without continuous aBL activation. HEPES was supplemented to the culture medium to maintain physiological pH during leachate collection under ambient atmosphere. Since bicarbonate‐buffered media rely on a controlled 5% CO2 environment to maintain pH, HEPES is routinely used as an additional buffer in experiments conducted outside CO_2_ incubators to prevent pH drift [64, 65]. After treating the cells for 24 h with the leachates, the leachates were aspirated off, and 0.5 mg/mL MTT in DI water was added and incubated at 37°C under a humidified atmosphere containing 5% CO_2_ / 95% air for 3 h. Within 3 h, metabolically active cells formed formazan crystals that were dissolved in DMSO for up to 30 min at room temperature. Finally, the absorbance at 590 nm and 650 nm was measured using a microplate reader (BioTek Cytation 5), and the relative cell viability (RCV) was determined using Equation (2).

(2) $$\mathrm{RCV}\;\left(\%\right)=\frac{\left({\mathrm{Abs}}_{\mathrm{sample},590\;\mathrm{nm}}-{\mathrm{Abs}}_{\mathrm{sample},650\;\mathrm{nm}}\right)}{\left({\mathrm{Abs}}_{\mathrm{untreated},590\;\mathrm{nm}}-{\mathrm{Abs}}_{\mathrm{untreated},650\;\mathrm{nm}}\right)}\;\times \;100$$

### 5.13 In Vitro Hemocompatibility Assessment

#### 5.13.1 Collection and Processing of Porcine Whole Blood

All procedures pertaining to the use of porcine whole blood (WB) were approved by the Institutional Animal Care and Use Committee of the University of Georgia. Porcine WB was collected in 3.2% sodium citrate using syringes via blind draws from the donor population. Collected WB was analyzed for hematology parameters (via Element HT5 Veterinary Analyzer) and separated into platelet‐rich plasma (PRP) through centrifugation at 280 xg for 12 min. The observed platelet count was analyzed through the Element HT5 Veterinary Hematology Analyzer. All blood processing and testing were initiated within 2 hours of the initial draw.

#### 5.13.2 Hemolysis

Hemolytic activity of RSNO‐coated CIs and controls was assessed in accordance with ISO 10993‐4 guidelines. Fresh porcine whole blood (WB) was collected, and baseline plasma hemoglobin levels were quantified using an Element HT5 Veterinary Hematology Analyzer. The blood was then diluted in CMF‐PBS to obtain a hemoglobin concentration of 10 mg/mL. Samples (3 cm in length) were placed into 2 mL of diluted WB in 2 mL microcentrifuge tubes and incubated at 37°C for 3 h with manual rocking every 30 minutes. Diluted WB in CMF‐PBS and DI water were used as a blank reference and positive control, with HDPE evaluated as the negative control. Following incubation, the samples were centrifuged at 800 g for 15 min to separate intact red blood cells. The resulting supernatants were reacted with Drabkin's reagent at a 1:1 ratio for 15 min, and absorbance was measured at 540 nm to quantify released hemoglobin. Hemolysis percentages were calculated using Equation (3).

(3) $$\mathrm{Hemolysis}\;\left(\%\right)=\frac{\left({\mathrm{Abs}}_{\mathrm{sample}}-{\mathrm{Abs}}_{\mathrm{blank}}\right)}{\left({\mathrm{Abs}}_{\mathrm{positive}\;\mathrm{control}}-{\mathrm{Abs}}_{\mathrm{blank}}\right)}\;\times \;100$$

#### 5.13.3 Platelet Adhesion

Platelet adhesion on RSNO‐coated CIs and control surfaces was evaluated using an established protocol with slight modifications [16]. Platelet‐rich plasma (PRP) was combined with CMF‐PBS to achieve a final platelet concentration of 2 × 10^8^ platelets mL^−1^, and coagulation was restored by adding calcium chloride (2.5 mM). Samples (3 cm in length) were exposed to 2 mL of the diluted PRP. Incubation was carried out for 90 min at 37°C with manual rocking every 30 min to promote platelet interaction with the surfaces. Following incubation, samples were carefully cut to 1.2 cm in length and rinsed with calcium‐ and magnesium‐free phosphate‐buffered saline (CMF‐PBS) to remove loosely associated platelets. Platelets remaining on the surfaces were lysed with 2% (v/v) Triton X‐100 in CMF‐PBS and quantified by lactate dehydrogenase (LDH) assay (Roche Cytotoxicity Detection Kit). Absorbance was measured at 492 nm with a reference wavelength of 620 nm, and platelet numbers were calculated using a calibration curve generated from lysed platelet standards.

### 5.14 Ethylene Oxide Sterilization

Control and SNAT‐coated CIs were placed in a Crosstex Duo Check sterilization indicator bag to verify successful sterilization before being transferred to a sterilization chamber containing an ethylene oxide (EtO) sterilizing gas ampoule. The ampoule was activated by cracking it, and the chamber was sealed for 12 h to complete the sterilization cycle, followed by a 2‐h purge cycle. Sterilized samples were subsequently placed in a desiccator for 2 h prior to further evaluation.

### 5.15 In Vivo Antibacterial Evaluation of SNAT Catheter Inserts

The antibacterial performance of the SNAT‐containing catheter inserts (SNAT CIs) was evaluated in vivo using a well‐established rabbit central venous catheter model [66]. The SNAT CIs were fabricated as described in Section 5.4. However, the fibrance fibers were cut to 15 cm segments. The fibers were inverted every 5 coats and allowed to dry for 2 min. After 20 coats, fibers were dried overnight, trimmed to 10 cm, topcoated with RTV, and dried overnight once more. The SNAT CIs were further dried in a dessicator for 1 hour and EtO sterilized. All animal procedures were approved by the University of Georgia Institutional Animal Care and Use Committee (IACUC; AUP A2023 11–012‐A4) and conducted in accordance with institutional and federal guidelines. Eight sexually intact white male New Zealand rabbits (2.7–3.5 kg) were used in this study. Animals were initially premedicated with intramuscular injections of xylazine (3 mg kg^−^^1^), ketamine hydrochloride (10 mg kg^−1^), and buprenorphine (0.03 mg kg^−1^), followed by maintenance anesthesia using isoflurane (1–4%) and 100% oxygen using a facemask. Lactated Ringer's solution was administered (10 mL kg^−1^ h^−1^) through an intravenous catheter in the marginal ear vein throughout the procedure.

Following aseptic preparation of the surgical site of the ventral cervical region, a subcutaneous injection of lidocaine (2 mg kg^−^^1^) was administered along the site of the incision. Approximately a 6 cm midline incision was made from the larynx to the manubrium. A new airway was established by performing tracheostomy and placing a cuffed endotracheal tube within the trachea; the airway circuit was then transferred and maintained on a small animal ventilator. The tracheostomy site was covered using sterile gauze to maintain sterility for the remainder of the surgery. Through the same incision, the right external jugular vein and its facial vein branch were isolated for catheter placement. For antibacterial evaluation, EtO pre‐sterilized control and SNAT catheter inserts (10 cm) were incubated with *S. aureus* (OD_600_ = 0.2; ∼10^7^ CFU mL^−1^) for 1 h prior to implantation. The catheter inserts were introduced through a venotomy in the facial vein and advanced approximately 6 cm into the cranial vena cava before being secured with sterile silk sutures. For the duration of the implantation period, the skin incision was temporarily closed in a single layer, leaving a small gap for the endotracheal tube and its circuit connection to be maintained, and the surgical site was covered with a sterile drape. This implantation approach preserved blood flow through the external jugular vein over a 7‐h implantation period. For SNAT CIs, aBL irradiation was applied throughout implantation.

At the conclusion of the study, animals received sodium heparin (330 IU kg^−^^1^) intravenously 5 min prior to euthanasia to minimize post‐mortem thrombosis. Animals were euthanized with sodium pentobarbital (200 mg kg^−^^1^, Euthasol, Virbac). The catheter insert and the vascular tissue it was inserted into were then explanted and sectioned into subcutaneous, jugular, and suture‐associated regions for thrombus and bacterial analyses. Surrounding tissue samples were also collected for microbiological evaluation.

#### 5.15.1 Ex Vivo Analysis

Following explantation, ex vivo evaluation of the CIs was performed. The jugular and facial veins were transected proximal to the CI, and the jugular vein was transected distal to the catheter tip. The vein was then opened longitudinally to expose and carefully remove the CI. To assess antibacterial efficacy in vivo, viable bacteria associated with the catheter inserts and surrounding tissue were quantified. Sections from the subcutaneous, jugular, and suture‐associated regions of each CI were measured, while adjacent tissue samples were weighed prior to processing. Samples were homogenized (OMNI TH homogenizer, Kennesaw, GA) and vortexed in 1 mL sterile PBS for 1 min each. Homogenates were serially diluted in sterile PBS and plated on LB agar. Following incubation at 37°C for 24 h, bacterial viability was quantified as colony‐forming units normalized to catheter surface area (CFU cm^−2^) or tissue mass (CFU mg^−1^).

### 5.16 Statistical Analysis

All data are presented as mean ± standard deviation (SD). Statistical differences among sample groups and across time points were evaluated using one‐way or two‐way ANOVA followed by Tukey's post hoc test for multiple comparisons. Outliers were determined via Grubbs' test with a significance level of *p* < 0.05 for NO release and in vitro bacteria studies. The sample size (n) was mentioned in the results section for each experiment. All analyses were performed using Origin software (Northampton, MA, USA). Unless otherwise stated, differences were considered statistically significant at *p* < 0.05 for all statistical analyses.

## Author Contributions

**Alexander Bruckmann**: conceptualization; data curation; formal analysis; investigation; methodology; visualization; writing – original draft preparation; writing – review & editing. **Adam Brooks Goodman**: conceptualization; data curation; formal analysis; investigation; methodology; visualization; writing – original draft preparation; writing – review & editing. **Myddelton C. Parker**: methodology; investigation; formal analysis**;** investigation**;** writing – original draft preparation; writing – review and editing. **Mark Garren**: formal analysis; methodology; writing – original draft preparation; writing – review and editing. **Ji Eun Park**: Investigation**;** writing – review and editing. **Chad Schmiedt**: resources; supervision; writing – review and editing. **Hitesh Handa**: methodology; resources; supervision; funding acquisition; writing – review and editing. **Elizabeth J. Brisbois**: conceptualization; funding acquisition; methodology; project administration; resources; supervision; writing – review and editing.

## Funding

The authors acknowledge financial support from the National Institutes of Health (NIH), including the National Heart, Lung, and Blood Institute (NHLBI) under Award Numbers R01HL170574, and the National Center for Advancing Translational Sciences (NCATS) under Award Numbers UL1TR002378 and KL2TR002381.

## Ethics Approvals

All animal procedures were approved by the University of Georgia Institutional Animal Care and Use Committee (IACUC; AUP A2023 11–012‐A4) and conducted in accordance with institutional and federal guidelines. A detailed description of the animal model can be found in “In vivo antibacterial evaluation of SNAT catheter inserts.”

## Conflicts of Interest

Drs. Hitesh Handa and Elizabeth Brisbois are the founders of a startup company that explores the possibilities of using nitric oxide‐releasing materials for medical applications.

## Supporting information




**Supporting File**: advs77846‐sup‐0001‐SuppMat.docx.

## Data Availability

The data that support the findings of this study are available from the corresponding author upon reasonable request.
