# Supplementary material for: Smartphone‐Guided Visible Light Modulation of RSNOs for Antimicrobial Activity via Controlled Nitric Oxide Release
Source: Adv Sci (Weinh). 2026 Sep 27:e77846. Online ahead of print. doi: 10.1002/advs.77846 (PMC13616380; doi:10.1002/advs.77846)
Supplement: Supplementary file 1 [file ADVS-9999-e77846-s001.docx]

**Smartphone-Guided Visible Light Modulation of RSNOs for Antimicrobial Activity via Controlled Nitric Oxide Release – Supporting Information**

**Alexander Bruckmann^1†^, Adam Brooks Goodman^1†^, Myddelton C. Parker^1^, Mark Garren^1^, Ji Eun Park^3^, Chad Schmiedt^3^, Hitesh Handa*^1,2^, Elizabeth J. Brisbois*^1^**

**^†^ Authors declare equal contributions**

^1^ School of Chemical, Materials, and Biomedical Engineering, College of Engineering, University of Georgia, Athens, Georgia, USA

^2^ Department of Pharmaceutical and Biomedical Sciences, College of Pharmacy, University of Georgia, Athens, Georgia, USA

^3^ Department of Small Animal Medicine and Surgery, College of Veterinary Medicine, University of Georgia, Athens, Georgia, USA

Prof. Elizabeth J. Brisbois and Hitesh Handa

School of Chemical, Materials, and Biomedical Engineering

College of Engineering

University of Georgia

302 East Campus Rd

Athens, GA 30602

E-mail: [ejbrisbois@uga.edu](mailto:ejbrisbois@uga.edu), [hhanda@uga.edu](mailto:hhanda@uga.edu)

**Supporting Methods:**

1.1 Apparent quantum yield of NO release

The apparent quantum yield of NO release was calculated for each RSNO-coated CI following previously reported methods. The final 5 min (15–20 min) of each 20-min wavelength-dependent irradiation period were used for the calculation. NO generated during irradiation above the corresponding dark-state NO release was determined from the NO analyzer output. The number of NO molecules generated during photolysis was calculated using Equation 1.

| nNO = (Xlight – Xdark) x t x NOAconst | **(1)** |
| --- | --- |

where Xlight and Xdark represent the NO concentration measured during irradiation and under dark conditions, respectively, t is the irradiation time, and NOAconst is the instrument-specific conversion constant. The number of incident photons was calculated from the measured/manufacturer-reported optical power according to Equation 2.

| nPhoton = P x t x λ/(h x c x NA) | **(2)** |
| --- | --- |

where P is the optical power, t is the irradiation time, λ is the wavelength, h is Planck’s constant, c is the speed of light, and NA is Avogadro’s constant. The apparent quantum yield was subsequently calculated using Equation 3.

| Apparent quantum yield (%) = (nNO/nPhoton) x 100% | **(3)** |
| --- | --- |

The manufacturer-reported optical powers were 20 mW for red and blue light and 10 mW for green light.

1.2 Diffusion of RSNOs in physiological conditions

Diffusion of RSNOs from RSNO-coated CIs was evaluated with and without continuous blue light illumination over 24 h under physiological conditions. Samples (1 cm in length) were fully submerged in 2 mL of PBS with EDTA within 2 mL microcentrifuge tubes and incubated at 37 °C. At designated time points (6, 12, and 24 h) the storage buffer was extracted and measured at 340 nm, where RSNOs have a stronger absorbance maxima compared to the weaker π→π* transition at ~590 nm,^1, 2^ using an UV–vis spectrophotometer (Cary 60, Agilent Technologies). Fresh PBS was replaced in each tube at each time point. The absorbance values collected were used to determine the concentration of RSNO diffusing into the buffer over time. The molar absorptivity values of SNAP and GSNO in PBS with EDTA at 340 nm were determined to be 1060 and 785 M^-1^ cm^-1^, respectively. The lipophilic nature of SNAT and Ph_3_-CSNO prevented their dissolution in PBS with EDTA, preventing the determination of molar absorptivity values.

1.3 Staphyloxanthin (STX) extraction

The extraction of STX was adapted from previous reports.^3, 4^ In brief, 1 mL of *S. aureus* grown in 20 mL LB medium was subcultured into 20 mL Tryptic Soy Broth (TSB; Sigma Aldrich; Cat. 22092-500G) for 24 h at 37 °C and 150 rpm. The bacteria culture was centrifuged at 3000 xg for 10 min and washed with 10 mL sterile PBS. This washing step was repeated before 4 mL methanol was added. The methanol extraction was performed for 24 h at 37 °C and 150 rpm. The supernatant was collected and centrifuged at 4000 xg for 10 min to remove any residual particles. The resulting methanol extract was further concentrated via rotary evaporation adjusted to a volume of 1 mL prior to obtaining the UV-vis spectra (Cary 60, Agilent Technologies).

1.4 *In vitro* antibacterial evaluation of SNAT catheter inserts

Sterilized SNAT-coated CIs were evaluated for antibacterial activity against *S. aureus* under static conditions. Prior to testing, the catheter inserts were sterilized using EtO. Inserts were exposed to *S. aureus* (OD_600_ = 0.1; ~10^7^ CFU mL^-1^) and incubated in 2 mL PBS for 7 h under the designated treatment conditions: SNAT without aBL, aBL alone using an uncoated control insert, or the combination of SNAT and aBL. Following incubation, the inserts were removed from the tubes, transferred to 1 mL sterile PBS, and homogenized and vortexed for 1 min each to detach adhered bacteria. The resulting suspensions were serially diluted in sterile PBS and plated on LB agar. Following incubation at 37 °C for 24 h, bacterial viability was quantified as CFU cm^-2^.

**Table S1**: Apparent quantum yield of NO release for SNAP-, GSNO-, SNAT-, and Ph_3_-CSNO-coated catheter inserts following wavelength-dependent light irradiation. Values were calculated using the final 5 min of each 20-min irradiation period following previously reported methods (*n≥5*).

| RSNO | Red-Light Quantum Yield (%) | Green-Light Quantum Yield (%) | Blue-Light Quantum Yield (%) |
| --- | --- | --- | --- |
| GSNO | 0.25 (±0.24) | 3.93 (±1.23) | 6.27 (±2.77) |
| SNAP | 0.21 (±0.13) | 0.87 (±0.45) | 3.21 (±1.76) |
| SNAT | 0.31 (±0.50) | 2.52 (±2.63) | 3.10 (±1.61) |
| Ph_3_-CSNO | 0.19 (±0.17) | 1.59 (±1.08) | 1.65 (± 0.61) |

**Figure S1.** Diffusion of SNAP (**A**) and GSNO (**B**) from RSNO-coated fibers without and with antimicrobial blue light (BL) under physiological conditions over 24 h. Data are shown as mean ± SD (*n=3*).

**Figure S2.** UV-vis spectra of SNAT and Ph_3_-CSNO diffusion from RSNO-coated CIs under physiological conditions. Data are shown as mean ± SD (*n=3*).

**Figure S3.** Thermal imaging of SNAT-coated catheter inserts during continuous blue-light irradiation over 24 h. Representative thermal images were collected at 0, 2, and 24 h of irradiation, followed by measurement after 15 min at room temperature (*n=3*).Representative thermal images were collected at 0 **A)**, 2 **B)**, and 24 h **C)** of irradiation, followed by measurement after 15 min **D)** at room temperature (CI’s *n=3;* Control *n=1*).

**Figure S4.** Average surface flux of NO released from SNAP-coated CIs under physiological conditions over 24 h when irradiated with blue-, green, and red-light (BL,GL, and RL) in comparison to the control (CTRL). Blue-, green, and red-light samples were irradiated during each time point as well as during storage between measurements. Data are shown as mean ± SD (*n≥3*)

**Figure S5.** Average surface flux of NO released from GSNO-coated CIs under physiological conditions over 24 h when irradiated with blue-, green, and red-light (BL,GL, and RL) in comparison to the control (CTRL). Blue-, green, and red-light samples were irradiated during each time point as well as during storage between measurements. Data are shown as mean ± SD (*n≥3*)

**Figure S6.** Average surface flux of NO released from SNAT-coated CIs under physiological conditions over 24 h when irradiated with blue-, green, and red-light (BL,GL, and RL) in comparison to the control (CTRL). Blue-, green, and red-light samples were irradiated during each time point as well as during storage between measurements. Data are shown as mean ± SD (*n≥3*).

**Figure S7.** Average surface flux of NO released from CSNO-coated CIs under physiological conditions over 24 h when irradiated with blue-, green, and red-light (BL,GL, and RL) in comparison to the control (CTRL). Blue-, green, and red-light samples were irradiated during each time point as well as during storage between measurements. Data are shown as mean ± SD (*n≥3*).

**Figure S8.** Staphyloxanthin extraction. **A)** UV-vis absorbance was measured for 4,4′-diapophytoene (286 nm) , 4,4′-diaponeurosporene (435 nm), 4,4′-diaponeurosporenic acid (455 nm), and STX (465 nm) post extraction of two 20 mL TSB vials. **B)** *S. aureus* pellet after methanol extraction vs after centrifugation (control). **C)** Methanol extract post rotary evaporation (*n=1*).

**Figure S9.** Reduction of viable methicillin-resistant *S. aureus* **A)** and *C. albicans* **B)** for RTV, SNAP, GSNO, SNAT, and Ph3-CSNO CIs when irradiated with blue light (BL) compared to no light irradiation. Data are shown as mean ± SD (*n≥3*). Symbols signify p<0.05: * compared to RTV, # compared to RTV (BL), † compared to respective RSNO controls.

**Figure S10.** *In vitro* antibacterial activity of ethylene oxide sterilized SNAT-coated CIs against adhered *S. aureus* following 7 h of treatment with SNAT alone, aBL alone, or combined SNAT+aBL. Bacterial viability was quantified following recovery of adhered bacteria from the CIs. Data are shown as mean ± SD (*n=4*). * signifies p<0.05 compared to RTV controls.

**Figure S11**. Control of the Smart Module via Versalume app. The app connects to the Smart Module via Bluetooth. When connected, the light color and the intensity can be tuned (blue, green or red). The Smart module is capable to simulate all colors of visible light.

(1) Bartberger, M. D.; Houk, K. N.; Powell, S. C.; Mannion, J. D.; Lo, K. Y.; Stamler, J. S.; Toone, E. J. Theory, spectroscopy, and crystallographic analysis of *S*-nitrosothiols:: Conformational distribution dictates spectroscopic behavior. *J Am Chem Soc* **2000**, *122* (24), 5889–5890. DOI: DOI 10.1021/ja994476y.

(2) Williams, D. L. H. The chemistry of S-nitrosothiols. *Accounts Chem Res* **1999**, *32* (10), 869–876. DOI: DOI 10.1021/ar9800439.

(3) Yu, J.; Shen, L.; Yang, J.; Shi, J.; Huang, Y.; Shang, Y.; Yu, F. Staphyloxanthin-enriched extracts promote biofilm formation and oxidative stress resistance in Staphylococcus aureus. *Microbiology Spectrum* **2025**, *13* (12), e00996–00925.

(4) Dong, P. T.; Mohammad, H.; Hui, J.; Leanse, L. G.; Li, J.; Liang, L.; Dai, T.; Seleem, M. N.; Cheng, J. X. Photolysis of Staphyloxanthin in methicillin‐resistant Staphylococcus aureus potentiates killing by reactive oxygen species. *Advanced science* **2019**, *6* (11), 1900030.
